# Integrating bulk and single‐cell RNA sequencing reveals cellular heterogeneity and immune infiltration in hepatocellular carcinoma

**DOI:** 10.1002/1878-0261.13190

**Published:** 2022-03-01

**Authors:** Tingjie Wang, Ningxin Dang, Guangbo Tang, Zihang Li, Xiujuan Li, Bingyin Shi, Zhong Xu, Lei Li, Xiaofei Yang, Chuanrui Xu, Kai Ye

**Affiliations:** ^1^ School of Automation Science and Engineering Faculty of Electronic and Information Engineering Xi’an Jiaotong University China; ^2^ Genome Institute The First Affiliated Hospital of Xi’an Jiaotong University China; ^3^ School of Life Science and Technology Xi’an Jiaotong University Xi’an, Shaanxi China; ^4^ Department of Endocrinology The First Affiliated Hospital of Xi’an Jiaotong University China; ^5^ Guizhou Provincial People's Hospital Guiyang China; ^6^ School of Pharmacy Tongji Medical College Huazhong University of Science and Technology Wuhan China; ^7^ School of Computer Science and Technology Faculty of Electronic and Information Engineering Xi’an Jiaotong University China; ^8^ Faculty of Science Leiden University The Netherlands; ^9^ MOE Key Lab for Intelligent Networks & Networks Security Faculty of Electronic and Information Engineering Xi’an Jiaotong University China

**Keywords:** HCC, multiomics, single cell, transcript factor, tumor heterogeneity

## Abstract

Efficacy of immunotherapy in hepatocellular carcinoma (HCC) is blocked by its high degree of inter‐ and intra‐tumor heterogeneity and immunosuppressive tumor microenvironment. However, the correlation between tumor heterogeneity and immunosuppressive microenvironment in HCC has not been well addressed. Here, we endeavored to dissect inter‐ and intra‐tumor heterogeneity in HCC and uncover how they contribute to the immunosuppressive microenvironment. We performed consensus molecular subtyping with non‐negative matrix factorization (NMF) clustering to stratify the inter‐heterogeneity profile of HCC tumors. We grouped HCC tumors from the Cancer Genome Atlas (TCGA) patients into three subtypes (S1, S2 and S3), where S1 was characterized as a ‘hot tumor’ profile with high expression level of T cell genes and rate of immune scores. S2 was characterized as a ‘cold tumor’ profile with the highest tumor purity score, and S3 as an ‘immunosuppressed tumor’ profile with the poorest prognosis and a high expression level of immunosuppressive genes such as cytotoxic T‐lymphocyte‐associated protein‐4, *TIGIT,* and *PDCD1*. Moreover, we combined weighted gene co‐expression network analysis and single‐cell regulatory network inference and clustering (SCENIC) in the single‐cell dataset of the S3‐like subtype (CS3) and identified a transcription factor, *BATF*, which could upregulate immunosuppressive genes. Finally, we identified a cell interaction network in which a myeloid‐derived suppressor cell‐like macrophage subtype could promote the formation of immunosuppressive T‐cells.

## Graphical abstract

By integrating bulk and single‐cell RNA sequencing in this study, we defined a 108‐gene classifier and investigated cellular heterogeneity, as well as immune infiltration in hepatocellular carcinoma (HCC). Patients with a high level of cytotoxic T‐lymphocyte‐associated protein‐4, TIGIT and LAG3 showed the poorest prognosis in both the Cancer Genome Atlas and International Cancer Genome Consortium cohorts. Additionally, single‐cell RNA sequencing analysis revealed high expression levels of these immunosuppressive genes and poor patient survival within the CS3 cluster. Our research indicated that BATF could be regulating the expression of immunosuppressive genes in HCC.

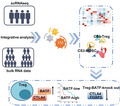

**Abbreviations**
aNK: activated NK cells
aT: activated T cells
CCA: cholangiocarcinoma
CNV: copy number variation
*CTLA4*
: cytotoxic T‐lymphocyte‐associated protein‐4
*CTNNB1*
: cadherin‐associated protein beta 1
End: endothelial cells
GSEA: gene set enrichment analysis
GSVA: gene set variation analysis
H1: hepatocyte cell cluster 1
H2: hepatocyte cell cluster 2
H3: hepatocyte cell cluster 3
H4: hepatocyte cell cluster 4
HCC: hepatocellular carcinoma
HCV: hepatitis C virus
ICC: intrahepatic cholangiocarcinoma
ICGC: International Cancer Genome Consortium
L‐R: ligand‐receptor
mB: memory‐like B cells
MDSC: myeloid‐derived suppressor cells
mMφ: MDSC‐like macrophages
mT: memory T cells
myCAF: myofibroblast cells
nB: naïve B cells
NMF: non‐negative matrix factorization
PD‐1: programmed cell death protein‐1
pT: proliferation T cells
SCENIC: single‐cell regulatory network inference and clustering
TAM‐Mφ: TAM‐like macrophages
TCGA: The Cancer Genome Atlas
Tem: effector memory T cell
TF: transcription factor
Tregs (tT): regulatory T cells
WGCNA: weighted correlation network analysis

## 1 Introduction

Hepatocellular carcinoma (HCC) is the most common form of primary liver cancer, with a 5‐year survival rate of 12–18% [1]. HCC is characterized by both inter‐ and intra‐tumor heterogeneity. Uncovering the molecular mechanisms underlying HCC heterogeneity is critical for the development of targeted therapies [2]. In recent years, genome‐wide analyses of mRNA expression profiles of large cohorts of HCC samples have been conducted [3]. However, most studies have focused on bulk RNA sequencing profiles, hindering the investigation of intra‐ and inter‐tumor heterogeneity.

To date, several medicines have been approved for HCC treatment with unsatisfactory results. The emergence of drug resistance in HCC treatment is inevitable due to tumor heterogeneity. Over 50% of HCC patients are currently administered systemic chemotherapies proven to be barely effective and toxic to the remaining normal liver [4]. In particular, anti‐cancer immunotherapy inhibiting programmed cell death protein‐1 (*PD‐1*)*/PD‐L1*, cytotoxic T‐lymphocyte‐associated protein‐4 (*CTLA4*), and various immune cell therapies, as well as vaccines, have sparked interest in the application of immuno‐therapeutics to HCC [5].

The effect of immunotherapy in HCC is reconciled by overall immune infiltration and enriched co‐occurrence of immune subpopulations [6]. Complex cell composition and characteristics in the tumor microenvironment (TME) highlight the presence of multiple non‐redundant mechanisms of cancer immune suppression. To improve immunotherapy efficacy, we should further investigate the heterogeneity of immune cells in different patient subtypes and identify suitable patients for specific immunotherapy. We should also study the mechanisms of tumor suppression and enrich immunotherapy efficacy. In recent years, single‐cell RNA sequencing (scRNAseq) has emerged as a powerful tool for revealing the heterogeneity of cells in the tumor immune microenvironment. For example, It has been reported that intrahepatic cholangiocarcinoma (CCA) could interact with regulatory T cells (Treg) through the ligand‐receptor (L‐R) pair of TIGIT‐PVR, leading to immunosuppression in intrahepatic CCA (ICC) [7, 8]. It has been reported that exhausted CD8^+^ T and Treg cells are preferentially enriched and potentially clonally expanded in HCC [9]. However, the correlation and consistency between inter‐ and intra‐tumor heterogeneity have not been evaluated.

In this study, we integrated analysis of scRNAseq and multi‐omics data unravel the tumor heterogeneity and immunosuppressive mechanism in HCC. The findings could facilitate clinical diagnosis and enrich HCC immunotherapy.

## 2 Materials and methods

### 2.1 Bulk RNA and single‐cell datasets

All the datasets applied in this study are listed in Table S1.

### 2.2 Bulk RNA data preprocessing

HCC bulk RNA data were retrieved from the Cancer Genome Atlas (TCGA; https://www.cancer.gov/tcga) and International Cancer Genome Consortium (ICGC; https://www.icgc‐argo.org), through GDC API, respectively. Samples without complete survival or clinical information were removed. We obtained 353 samples from TCGA and 232 samples from ICGC for subsequent analysis.

### 2.3 Identification of HCC subclasses

Genes with a mean absolute deviation of > 1 top genes were chosen for NMF clustering [10]. Subsequently, unsupervised NMF clustering methods were performed on the normalized expression data using the NMF r package [11]. The values of *k* when the magnitude of the cophenetic correlation coefficient began to fall were chosen as the optimal number of clusters [12].

### 2.4 Multi‐omics data acquisition and processing

Somatic mutation data of all HCC patients from the ‘Masked Somatic Mutation’ category in TCGA were processed using varscan software (https://portal.gdc.cancer.gov/). Mutations were analyzed and visualized using maftools (version 2.10.0) [13]. Enrichment scores of the hallmark genes were evaluated using single‐sample gene set enrichment analysis (GSEA, ssGSEA) using the gene set variation analysis (‘GSVA’) r package (version 1.42.0) [14]. The hallmark gene sets were obtained from MSigDB database from GSEA software (http://www.gsea‐msigdb.org/gsea/downloads.jsp).

### 2.5 Differentially expressed gene analysis

The ‘Limma’ package was used to perform the differentially expressed gene (DEG) analysis. An empirical Bayesian method was applied to estimate the differential genes between two clusters identified by the NMF clustering method using moderated *t*‐tests [15]. Considering the high immune cell infiltration in S1 and S3, and high tumor purity in S2, we then overlapped the differentially expressed genes in S1 and S3 with immune genes from ImmPort (https://immport.niaid.nih.gov/home) and excluded the immune genes in S2 to obtain the final candidate genes. The adjusted *P*‐value for multiple testing was calculated using the Benjamini–Hochberg correction. Genes with an absolute log_2_ fold change greater than one and FDR < 0.05 were identified as signatures between two clusters. We performed differential analysis for each cluster, which was compared with both of the other two clusters to select either significantly upregulated (log_2_FC > 1; FDR < 0.05) or significantly down‐regulated (log_2_FC < −1; FDR < 0.05) genes.

### 2.6 Estimation of immune infiltration and tumor purity

We downloaded the ‘CIBERSORT’ scripts (https://cibersort.stanford.edu/) to estimate the immune composition of HCC patients using the normalized express matrix, and the patients whose *P*‐value was < 0.05 were adopted in the immune infiltration [16]. Immune, stromal and tumor purity scores were calculated using the ‘Estimate’ r package [17].

### 2.7 Single‐cell RNAseq data processing

The raw gene expression matrix was imported and processed using the Seurat r package (version 3.1.2) [18]. Single‐cell datasets were downloaded from the Gene Expression Omnibus dataset (Table S1). Cells with UMI counts < 200 were removed. Library size normalization was performed in each group on the raw matrix to obtain the normalized counts via Seurat (version 3.1.2). We then applied the mean‐dependent trend method in the Scran package (version 1.10.1) to identify highly variable genes [19]. Significant genes (FDR ≤ 1e−3) were selected for principal component analysis (PCA) to reveal biologically meaningful variations. The number of components used was determined based on the JackStraw function. Clusters were computed using the FindClusters function (resolution = 0.8). Clusters were visualized using uniform manifold approximation and projection (UMAP) as implemented in Seurat. Differential expression between clusters was calculated using a likelihood‐ratio test for single‐cell gene expression implemented in Seurat at a family‐wise error rate of 5%. Cell types were defined according to lineage‐specific marker genes. The batch effect was removed using CCA.

### 2.8 Enrichment score

The enrichment score was calculated to evaluate subtype distribution in each cluster. First, we calculated the frequency of each subtype in each cluster [20]. Then, we divided the gP by cluster frequency (cell number in the cluster divided by total cell number) and obtained the enrichment score of each subtype in every cluster.

### 2.9 Co‐expression network construction

A normalized expression matrix was used to construct a weighted co‐expression network (WGCNA) using the r package (1.69) [21]. To attenuate the effects of noise and outliers, the analyses were performed on pseudocells, calculated as averages of 10 cells randomly chosen within each cell type [22]. A co‐expression network was constructed using the blockwiseModules function with default parameters. Correlation between module eigengenes and cell‐type information determined the significance of modules using Pearson's test. Afterward, the hub genes were selected based on each gene’s modular connectivity and phenotypic trait relationship in the hub module. Module connectivity was defined as the absolute value of Pearson's correlation between genes (module membership). The clinical trait relationship was defined as the absolute value of Pearson's correlation between each gene and cell type (gene significance). We set the module membership at > 0.7 and the gene significance at > 0.6 for candidate hub genes.

### 2.10 SCENIC analysis

We used the R package SCENIC (https://github.com/aertslab/SCENIC, version 1.1.1‐10, RcisTarget 1.6.0, and AUCell 1.8.0) to analyze the enrichment of transcriptome factors in cell subtypes [23]. The input matrices for each sample in SCENIC were the raw UMI counts from Seurat. We kept genes with a sum of expression > 3 × 0.005 × cell numbers detected in at least 0.5% of the cells. Following the standard SCENIC procedure, we used GENIE3 method (for a single sample) and GRNBoost (for the combined sample) to identify potential transcription factor (TF) targets. In addition, the activity of each regulon in each cell was evaluated using AUCell, which calculates the area under the recovery curve and integrates the expression ranks across all genes in a regulon.

### 2.11 Statistical analysis

All computational and statistical analyses were performed using the r software (https://www.r‐project.org/). The unpaired Student's *t*‐test was used to compare two groups with normally distributed variables, and the Mann–Whitney *U*‐test was used to compare two groups with non‐normally distributed variables. Survival analysis was performed using the ‘survival’ r package. An optimal cutoff value defining two groups of patients with different survival curves was determined using the program X‐tile [24]. The log‐rank test was used to determine whether the survival curves were significantly different.

### 2.12 Immunosuppressed score, liver score and activated T cells scores

We adopted the expression of repressed T marker genes as well as liver marker genes to further calculate the immunosuppressed score and liver score. Liver score was calculated as the average expression of 24 liver marker genes from Kim et al. [25] (Table S7). Likewise, immunosuppressed score and activated T cell score were defined based on 35 known repressed markers and 28 activated GZMK‐CD8 genes from Guo et al. [26] (Table S7).

## 3 Results

### 3.1 Non‐negative matrix factorization identifies three subtypes in HCC

**Fig. 1:**
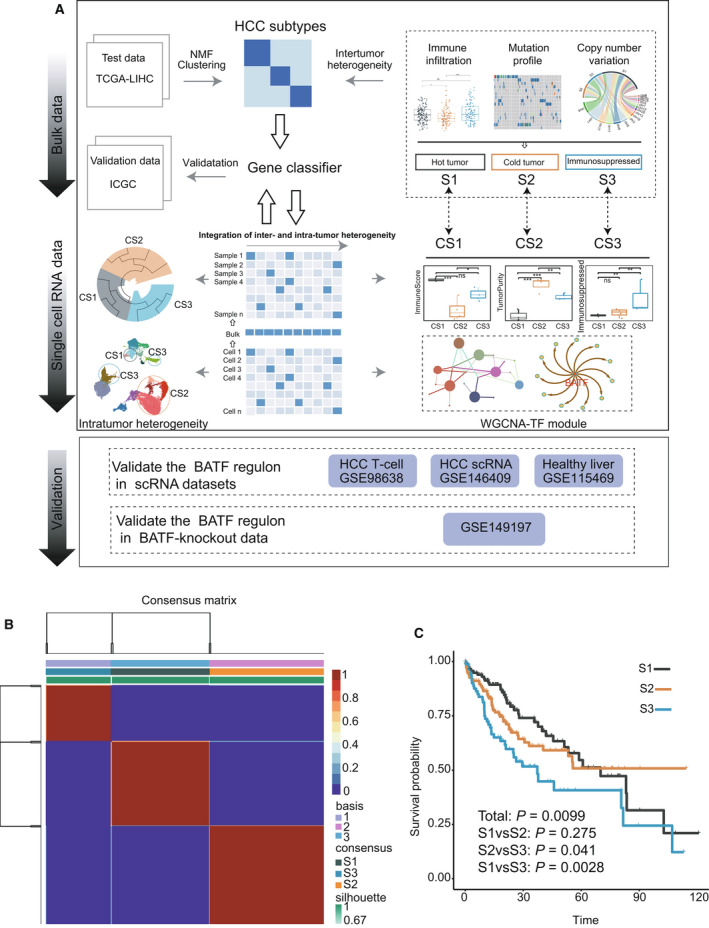
Identification of HCC subclasses using NMF consensus clustering. (A) Overview of the study design. We first classified the patients into three subtypes in TCGA LIHC cohort via NMF clustering method, and analyzed their inter‐tumor heterogeneity including immune infiltration status and mutation profile. We defined the subtype S1 as ‘hot tumor’ with the high immune infiltration score, S2 as ‘cold tumor’ with high tumor purity score, and S3 as ‘immunosuppressed tumor’ with a high level of immune‐repressed score. Patients in immunosuppressed subtype S3 exhibited the poorest prognosis. We then construed a 108‐gene classifier by integrating bulk RNA data with scRNAseq data using the differential genes from the three HCC subtypes. The scRNAseq samples could also be grouped into the corresponding HCC subtypes (S1‐CS1, S2‐CS2, S3‐CS3) using the 108‐gene classifier. Subsequently, we investigated the intra‐tumor heterogeneity in the three subtypes. Of note, we found that transcript factor *BATF* could promote the expression level of immune‐repressed genes such as *CTLA4* and *TIGIT,* and we verified the *BATF* regulon in the BATF‐knock out cell line data and other two single‐cell datasets. In this study, we demonstrated that *BATF* could promote the immunosuppressed genes and induce the poorest clinical prognosis in HCC. (B) Heatmap plot showing the consensus matrix of NMF clustering results using the gene expression data in TCGA LIHC cohort, colored by three HCC subtypes. (C) Overall survival curves showing the prognosis result of the three subtypes (S1, S2 and S3) obtained from NMF clustering in the TCGA LIHC cohort. Statistical significance was calculated using the log‐rank test (S1:120, S2:144, S3:89).

A schematic of the study design is shown in Fig. 1A. First, we obtained the transcriptome and somatic mutation profiles of 353 HCC patients from TCGA. We utilized consensus clustering analysis of the NMF algorithm and identified three distinct modification pattern clusters, including 120 cases in pattern cluster S1, 144 cases in cluster S2 and 89 cases in cluster S3. The heatmap of the consensus matrix exhibited sharp boundaries, indicating the accuracy and robustness of the clustering results (Fig. 1B, Fig. S1A). We also validated the clustering result in ICGC cohort and did the replication analysis using the SubMap (http://genepatern.broadinstitute.org/, Fig. S1B–E). Interestingly, we found that patients in S3 had the worst prognosis among the three subtypes (Fig. 1D). Meanwhile, we analyzed the heterogeneity of the clinical indicators in these three subtypes and found that patients in S3 had a significantly higher tumor stage (AJCC‐T3/T4 and Neoplasm disease stage III and IV; Table S2), which might partially explain the poor prognosis of this subtype.

### 3.2 Inter‐tumor TME heterogeneity in HCC

**Fig. 2:**
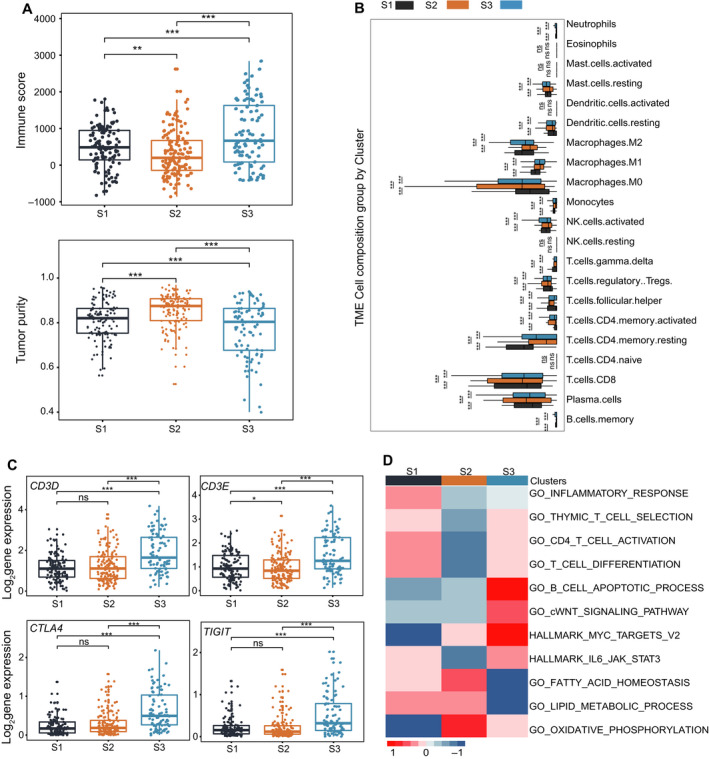
Investigation of the immunologic inter‐tumor heterogeneity in the three HCC subtypes. (A) Boxplots showing the immune and tumor purity scores in the three distinct malignant subtypes (S1:120, S2:144, S3:89; ns = no significance, *P < 0.05, **P < 0.01, ***P < 0.001). Pairwise comparison in the TCGA LIHC cohort was conducted by Wilcoxon rank‐sum test. For the boxplot, the centerline represents the median and box limits represent upper and lower quartiles. Each dot represents a sample. (B) Boxplots showing the 22 immune cell infiltrates ratio in the three distinct malignant subtypes in the significantly enriched patients (S1:27, S2:32, S3:61; ns, no significance, *P < 0.05, **P < 0.01, ***P < 0.001). Pairwise comparison was conducted by Wilcoxon rank‐sum test in the TCGA LIHC cohort. For the boxplot, the centerline represents the median and box limits represent upper and lower quartiles. (C) Comparisons of the gene expression level of immune genes in the three distinct malignant subtypes (S1:120, S2:144, S3:89; ns = no significance, *P < 0.05, **P < 0.01, ***P < 0.001). Pairwise comparison was conducted by Wilcoxon rank‐sum test in the TCGA LIHC cohort. In the boxplot, the centerline represents the median and box limits represent upper and lower quartiles. Each dot represents a sample. (D) We performed the GSVA analysis for the three HCC subtypes in the TCGA cohort, and made a pairwise comparison using the GSVA enrichment scores via Wilcoxon rank‐sum test. GSVA results show the heterogeneity of gene function in the three distinct malignant subtypes. Color indicates the GSVA enrichment score. Colors from blue to red indicate the GSVA score from low to high.

Next, we analyzed the heterogeneity of immune infiltration and tumor purity in the three subtypes using the ESTIMATE algorithm [17]. The results showed that the immune and stromal scores of S2 were significantly lower than those of S1 and S3 (*P* < 0.001), whereas S2 exhibited the highest tumor purity score (*P* < 0.001; Fig. 2A, Fig. S2A). Since S3 had the poorest prognosis among the three subtypes, we further characterized their immunologic landscape across the 22 immune‐related cell types with CIBERSORT (Section ‘Estimation of immune infiltration and tumor purity’). The results demonstrated that S1 had a higher abundance of activated NK cells (aNK), CD8^+^ T cells, M1 cells, and CD4 memory resting T cells but a lower abundance of Treg and M0 cells compared with S3. S2 had a lower abundance of M2 cells (Fig. 2B). In particular, T cell marker genes, such as *CD3E*, were highly expressed in S1 and S3 (Fig. 2C). We further investigated the profiles of immunosuppressed marker genes in the three subtypes and calculated the immunosuppressed score (Section ‘Estimation of immune infiltration and tumor purity’). Results indicated that immunosuppressed score (Fig. S2B) and marker genes such as *VEGFA, CTLA4, HAVCR2* and *TIGIT* were highly expressed in S3 (Fig. 2C, Fig. S2D). The high rate of immune‐repressive T cells might be correlated with a poor prognosis. In addition, patients in S3 had high levels of the macrophage marker gene *CD68* and EMT marker genes such as *MMP2* and *MMP9*, indicating that these cells in the tumor microenvironment may play important roles in tumor progression (Fig. S2D,E). In contrast, patients in S1 had the best prognosis result, with a higher level of activated T cell markers such as *CD3E, PRF1* and *GZMK* and exhausted marker genes of T cells such as *PDCD1* and *HAVCR2* than that in S2, as well as a lower immunosuppressed score than S3 (Fig. 2C, Fig. S2C,D). We also performed enrichment in GO_BP and HALLMARKER pathways using the differential genes in the three subtypes (Section ‘Enrichment score’) via GSVA to investigate the functional signatures intergroup. Results showed that immune‐related pathways, such as IL6‐STAT3 and thymic T cell selection, were enriched in S1 and S3, whereas CD4 activation, inflammatory response and T cell differentiation pathways showed a higher enrichment score in S1. In addition, higher enrichment scores of B cell apoptotic and WNT pathways were found in S3, compared with fatty acid, lipid metabolism and oxidative phosphorylation in S2 (Fig. 2D, Table S3). Taken together, S2 demonstrated features of ‘cold tumors’ due to a lower immune infiltration ratio, and S1 features of ‘hot tumors,’ while S3 showed ‘immunosuppressed tumors’ for the highly expressed immune‐repressive genes.

It has been reported that tumor genomic mutation is correlated with antitumor immunity [27]. We therefore analyzed frequency differences of somatic and copy number variation (CNV) mutations among HCC subtypes and distinct subtype‐specific mutation characteristics (Fig. 2A, detailed statistical analysis is shown in the Table S4). Specifically, S2 had a significantly higher frequency of cadherin‐associated protein beta 1 (*CTNNB1*) and *ARID1A* (45% and 11%, respectively) than S1 (14% and 4%) or S3 (9% and 4%). S3 exhibited higher frequency of *TP53* (49%) and *BAP1* (10%) compared with S1 (22% and 4%, respectively) and S2 (25% and 3%). Of note, we found that the immune score and expression of *CD3D* and *CTLA4* was higher in the *CTNNB1*‐non mutated group (Fig. S3B,C). It has been reported that a patient with *CTNNB1* mutation showed lower immune infiltration in HCC, and *ARID1A* mutation in ovarian clear cell carcinoma also has a role for immune inhibition [28, 29], which is consistent with our result. *BAP1* regulates cell death and mitochondrial metabolism [30], which is consistent with the higher expression level of exhausted marker genes in S3.

**Fig. 3:**
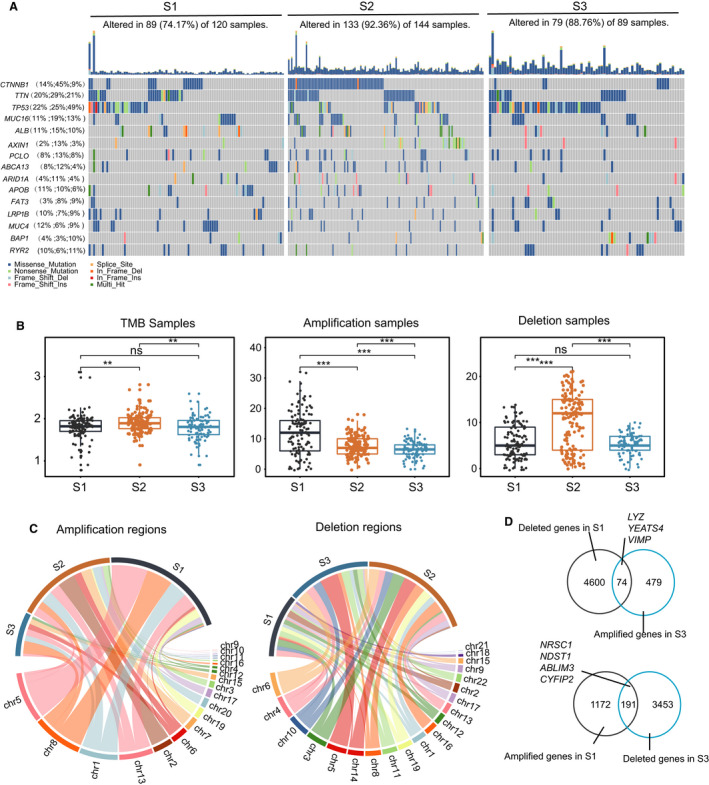
Investigation of the mutation profile inter‐tumor heterogeneity profiles in the three HCC subtypes. (A) The top 10 mutated genes in HCC across the three subtypes. The colors of rectangles in the body of the heatmap indicate different types of somatic mutations; the key identifying each mutation type is shown at the bottom below the color bar. The bar plot on the top shows the counts of mutations for each patient and the colors in the bar plots correspond to the colors showing mutation types in the body of the heatmap. The title of the heatmap showing the mutation sample number in each subtype includes amplification, missense mutations and deep deletions. The number on the left shows the gene mutation frequency in the three subtypes. (B) The number of mutations and copy number aberrations in the HCC subtypes in the TCGA LIHC cohort. Pairwise comparison was conducted by the Wilcoxon rank‐sum test (S1:120, S2:144, S3:89; ns, no significance, **P < 0.01, ***P < 0.001). In the boxplot, the centerline represents the median and box limits represent upper and lower quartiles. Each dot represents a sample. (C) Circos plot showing the amplification and deletion regions in the three subtypes. The width of the plot indicated the CNV mutation regions numbers in each subtype across the chromosomes. (D) Venn plots shows the genes amplified in S3 but deleted in S1 simultaneously (the first row), and the genes deleted in S3 but amplified in S1 simultaneously (the second row).

In addition, there was significant heterogeneity of the CNV profiles in the three subtypes. S1 had the most amplified variant samples, whereas S2 had the most deleted ones (Fig. 3B). In CNV mutated regions, patients in S1 were mainly amplified in the regions such as 1q, 5p, 8q, 6p and 11q, patients in S2 were amplified regions in 1q, 11q, 1q and 2q, and patients in S3 in S2, and 8q, and 13q (Fig. 3C, Fig. S4B). Remarkably, *YEATS4* and *VIMP*, which were highly expressed in S3, were deleted in S1 but amplified in S3 (Fig. 3D, Fig. S4A). *YEATS4* promotes HCC cell proliferation and colony formation [31]. *VIMP* inhibits cytokine production in human CD4^+^ effector T cells [32]. By contrast, *CYFIP2* and *ABLIM3*, which were highly expressed in S1, were simultaneously amplified in S1 but deleted in S3 (Fig. 3D, Fig. S4A). *CYFIP2* is highly abundant in CD4^+^ cells from multiple sclerosis patients and is involved in T cell adhesion, and *ABLIM3* is a component of adherent junctions with actin‐binding activity [33, 34]. Taken together, the highest mutation of *BAP1*, amplification of *YEATS4* and *VIMP*, and deletion of *CYFIP2* and *ABLIM3*, might induce an immune‐repressed environment in S3, while high mutation of *CTNNB1* might inhibit immune infiltration in S2.

### 3.3 A novel gene classifier obtained from integrating bulk and single‐cell transcriptomic data

**Fig. 4:**
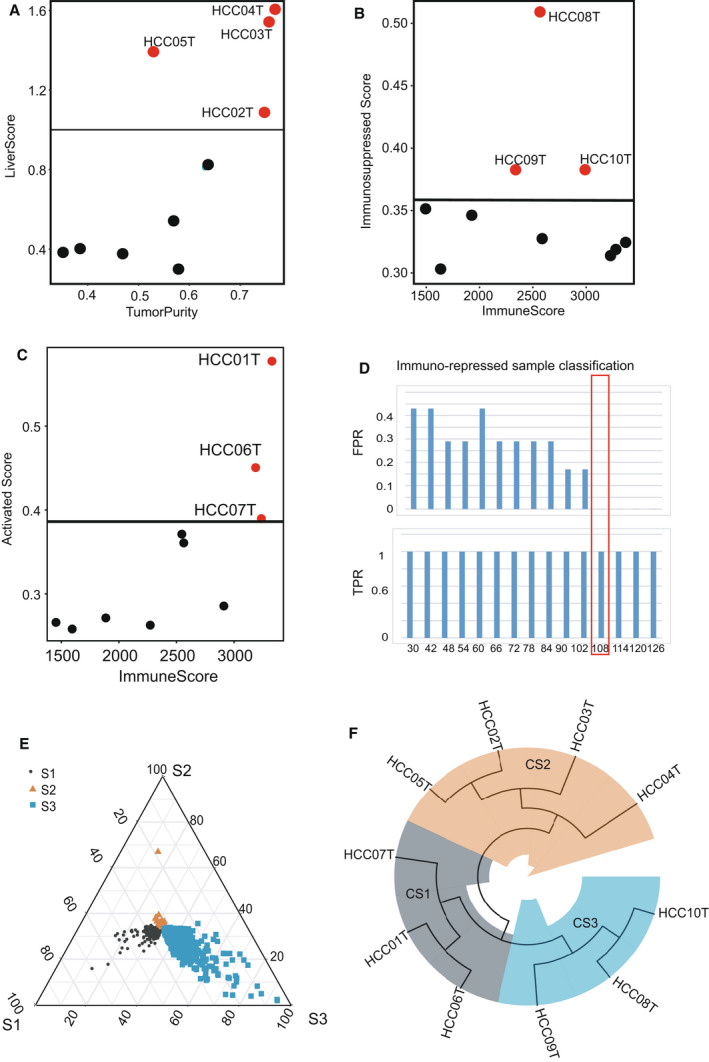
Integrated bulk RNA data with single‐cell data to obtain a gene classifier. (A) Scatter plot showing the distribution of liver scores calculated by the mean of 24 liver marker genes and tumor purity scores by ESTIMATE among the 10 bulked single‐cell RNAseq samples. The black horizontal line showing the positive cutoff value calculated by the upper quartile of liver scores. Red dot illustrates the positive tumor purity samples whose liver scores were higher than the cutoff value. (B) Scatter plot showing the distribution of immunosuppressed scores calculated by the mean of 35 repressed marker genes and immune scores by ESTIMATE among the 10 bulked single‐cell RNAseq samples. The black horizontal line showing the positive cutoff value calculated by the upper quartile of immunosuppressed scores. Red dot illustrates the positively immunosuppressed samples. (C) Scatter plot showing the distribution of aT scores calculated by the mean of 28 activated marker genes and immune scores by ESTIMATE among the 10 bulked single‐cell RNAseq samples. The black horizontal line shows the positive cutoff value calculated by the upper quartile of aT scores. Red dot illustrates the positively activated immune samples. (D) False‐positive rate (FPR) and true‐positive rate results in the immunosuppressed samples classification. (E) Ternary phase diagram showing the differential genes among the three HCC subtypes, colored and shaped by subtype (S1, S2 and S3). (F) Tree plot showing the hierarchical clustering result in the 10 single‐cell RNAseq samples, colored by subtype (CS1, CS2, CS3 in the cohort GSE149614).

To integrate scRNAseq and bulk RNA samples, we first construed a classifier reference set. Specifically, 10 scRNAseq samples from the GSE149614 dataset were initially bulked according to the mean expression values across the genes. Next, immunosuppressed, activated T cells (aT) and liver scores were calculated using the method given in Section onEstimation of immune infiltration and tumor purity’ and their upper quartile was defined as cutoff values of positive samples. Meanwhile, we evaluated immune and tumor purity scores of the 10 samples using ESTIMATE software. Among those 10 scRNAseq samples, we found that individual samples HCC02T, HCC03T, HCC04T and HCC05T possessed the highest liver and the lowest immunosuppressed scores (Fig. 4A), so they were defined as ‘cold tumor’ samples. In contrast, HCC08T, HCC09T and HCC10T had the highest immunosuppressed scores, indicating they were ‘immunosuppressed tumor’ samples (Fig. 4B). Likewise, HCC01T, HCC06T and HCC07T with high activated T cell scores were ‘hot tumor’ samples (Fig. 4C).

Next, we built a classifier by selecting the most specific genes to identify all the positive samples in the above reference set. We performed classification analysis in the reference set using series numbers of differentially expressed genes (30–126, sorted by log2‐fold change) obtained from the signatures in the three subtypes (S1, S2 and S3) (Section ‘Differentially expressed gene analysis’). The results showed that all false‐positive rates (FPRs) were 0 and all true‐positive rates (TPRs) were 1 when the top 108 genes were selected (Fig. 4D, Tables S5 and S6). When more genes were included, FPRs and TPRs values were not changed for classification of the reference set.

Next, we validated the classification effect in the TCGA and ICGC cohorts using a panel covering these 108 genes (Table S7). The samples were also grouped into three subtypes in the two large cohorts (Fig. S5A,B). S3 still had the poorest prognosis in TCGA (Fig. S5A) and had high expression levels of immunosuppressive genes, high immune scores, low tumor purity score, as well as the poorest prognosis (*CTLA4, TIGIT*; Fig. S5B,C,E,F) in ICGC. Moreover, the SubMap analysis among the subtypes obtained from the 108‐gene classifier validated the consistency between the TCGA and ICGC cohorts (Fig. S5D). The enriched pathways were also consistent between the two cohorts (Fig. S6). These results further confirmed that the 108‐gene classifier could map the single‐cell samples into the three subtypes and group the HCCs into the different immune status subtypes.

### 3.4 TME heterogeneity among the three HCC subtypes

**Fig. 5:**
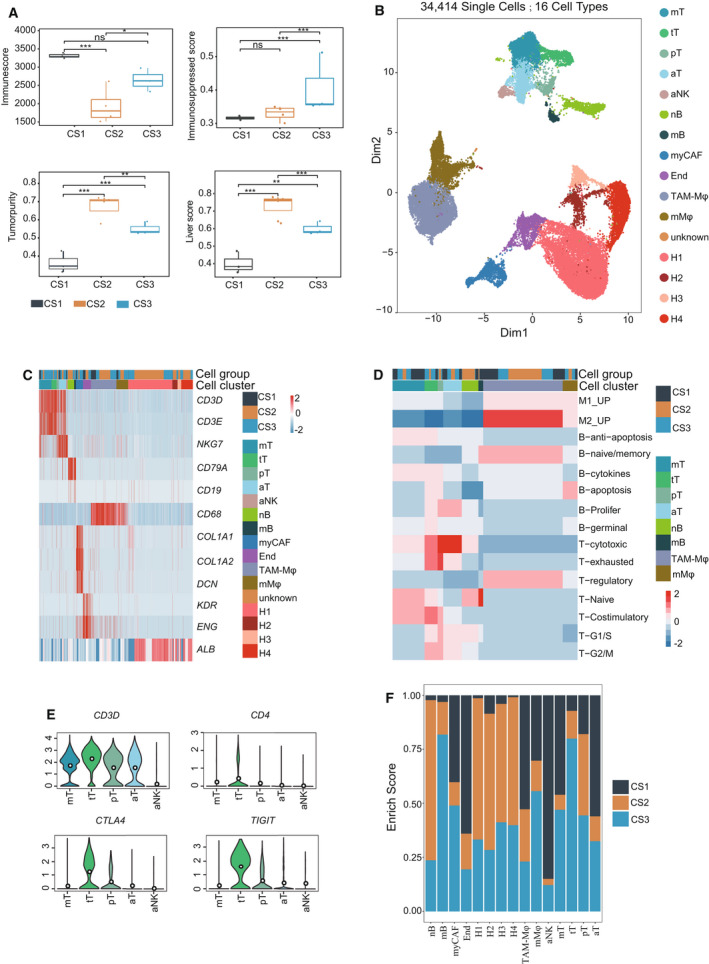
Investigation of the intra‐tumor heterogeneity of HCC. (A) Boxplot showing the immune, immunosuppressed, tumor purity and liver scores in the three subtypes in a single‐cell dataset (CS1:3, CS2:4, CS3:3; ns *P* > 0.05, *P < 0.05, **P < 0.01, ***P < 0.001). Pairwise comparison was conducted by Wilcoxon rank‐sum test in the single‐cell RNAseq cohort GSE149614). In the boxplot, the centerline represents the median and box limits represent upper and lower quartiles. Each dot represents a sample. (B) UMAP visualization of the 34 414 cells from 10 primary HCC tumor tissue patients in the single‐cell RNAseq cohort GSE149614. Different colors indicate distinct clusters. tT: treg T cells; pT: proliferative T cells. (C) Heatmap showing the expression of representative marker genes in the each cell type. Color key: Color gradient blue to red indicates relative expression levels from low to high. (D) The heatmap showing the GSVA enrichment result of immunological pathways among the immune subtypes (mT, tT, pT, aT, nB, mB, TAM‐Mφ and mMφ). Different colors indicate clusters and cell groups (CS1, CS2 and CS3). Color gradient blue to red indicates GSVA enrichment scores from low to high. (E) Violin plots show the gene expression level of immune genes in the single‐cell RNAseq cohort GSE149614 (aNK: 350, mT: 2763, tT: 1128, pT: 516, aT: 1600). In the violin plot, the centerline represents the median and box limits represent upper and lower quartiles; whiskers, data range. (F) Bar plot showing the cell distribution across the three subtypes CS1, CS2 and CS3, colored by subtype (CS1:3368; CS2: 15, 780; CS3: 15 266). The bar plot indicates that the immune‐repressed subtype tT (treg T cells) were enriched in immunosuppressed S3‐like subtype CS3.

We investigated regulatory mechanism of the immune‐repressive subtype and analyzed the heterogeneity of the tumor immune microenvironment at the cell level in the three single‐cell subtypes (CS1, CS2 and CS3). CS3 samples had the highest immune and immunosuppressed scores, and CS2 the highest tumor purity and liver scores (Fig. 5A). Therefore, CS1 was similar to S1, CS2 to S2, and CS3 to S3. Following gene expression normalization, dimensionality reduction, clustering and characterization based on cell lineage‐specific marker genes, these cells were grouped into 16 types, including 6007 T cells, 1845 B cells, 14 552 epithelial cells, 350 NK cells, 1850 endothelial cells and 1548 fibroblasts (Table S8, Fig. 5C). Then, four T cells, two B cells, and three macrophage sub‐cell types were obtained. Thereafter, the immune cells were classified by analyzing their gene expression profiles and functions via GSVA analysis using the immunological gene sets obtained from Chung et al. [35]. In T and NK cells, mT is a memory T cell type overexpressing genes such as *IL7R, CCR7* and *CD69*, as well as enrichment of naïve or mT signaling in GSVA. tT is an immune‐repressive T cell type with high expression levels of *HAVCR2, TIGIT, BATF* and *CTLA4* (Fig. 5D,E, Fig. S7A,B). T cells with high levels of *MKI67* and *TIGIT* were designated as proliferation T cells (pT) cells. Cytotoxic genes such as *PRF1* and *GZMA* were highly expressed in aT and NK cells, and *CD8A* was expressed in aT, indicating that they were effective CD8 T and NK cells, respectively (Fig. S7A). B cells enriched in either naïve or memory B cell signaling in GSVA and overexpression of *CCR7* and *CD69* were designated as memory‐like B cells (mB). Likewise, macrophages were divided into two subtypes: TAM‐like macrophages (TAM‐Mφ) with high expression levels of M2 signatures in GSVA, and TAM‐like genes, such as *APOE, C1QA, SLC40A1* and *GPNMB* (Fig. S7A,C), which was similar to the Mφ‐c2‐C1QA subtype in a previous report [36]. However, MDSC‐like macrophages (mMφ) showed both M1 and M2 signatures exhibiting high expression levels of pro‐inflammatory genes such as *FCN1* and *VCAN*, as well as the immune‐repressive gene *IL10* (Fig. S7A,C). The mMφ was similar to the Mφ‐c1‐THBS1 subtype reported previously, which is a myeloid‐derived suppressor cell (MDSC‐like) subtype [36]. In addition, CAF expressed high levels of αSMA (*ACTA2*), which are designated as myofibroblast cells (myCAF; Fig. S7C). The group distribution enrichment results showed that NK and aT cells were enriched in CS1, mT, mB, tT and mMφ, myCAF cells were enriched in CS3, and epithelial cells (H1, H2, H3, H4) were enriched in CS2, which further validates the classification effect of the gene classifier (Fig. 5F).

### 3.5 Transcription factor *BATF* and MDSC‐like macrophage cells could promote the formation of immunosuppressive cells

**Fig. 6:**
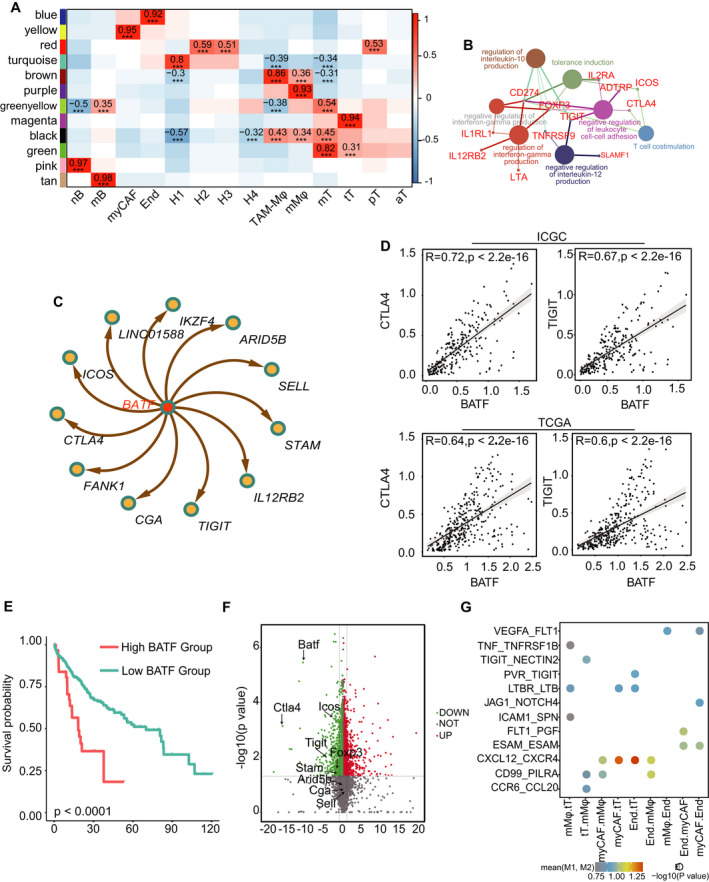
Investigation of the immunosuppression mechanism in single cells by combining WGCNA with SCENIC and cell interaction analysis. (A) WGCNA result demonstrated the correlation between the gene modules and cell types in single‐cell RNAseq cohort GSE149614. Columns were with cell types and rows with gene modules. Color gradient from blue to red indicates correlation between gene module and cell subtypes from low to high. The magenta gene module was positively correlated with immunosuppressive subtype tT, and the green module was correlated with memory T cell subtype mT (***P < 0.001). (B) Pathway network graph showing gene enrichment results in the tT subtype. Genes are indicated by red colors and pathways were indicated by the other colors. (C) TF‐regulon of *BATF* obtained from WGCNA‐TF analysis in immunosuppressed subtype tT (treg T cells), where the red node indicates TF and yellow nodes indicate target genes. (D) Scatter plot showing the correlation between log‐normalized expression of transcript factor *BATF* and the target genes such as *CTLA4*, *TIGIT* in the ICGC (first row) and TCGA (second row) cohorts (TCGA: 353; ICGC: 232; Pearson's correlation test). (E) Overall survival curves showing the prognosis result of critical TF *BATF* in TCGA cohort. Red and blue colors indicate patients with high and low *BATF* expression levels in the TCGA cohort, respectively. The cutoff was calculated by X‐tile. Statistical significance was calculated using the log‐rank test (BATF‐High: 36, BATF‐Low: 317). (F) Volcano plot showing the differential genes in the BATF‐knock out cell line RNAseq dataset (GSE149197) via Limma analysis, where a green color indicates downregulated genes (Log_2_FC < −1, FDR < 0.05) and red indicates upregulated genes (Log_2_FC > 1, FDR < 0.05) (upregulated genes: 1266, downregulated genes: 1347). *CTLA4, TIGIT* and *FOXP3* were all significantly downregulated in the BATF‐knock out data. (G) Dot plot showing the correlation of L‐R pairs among TME cell types via permutation test by CellPhoneDB: rows represent the L‐R pairs and columns represent cell type pairs. The color gradient from blue‐black to red indicates interaction scores of L‐R pairs from low to high, and circle size indicates significant *P*‐value of the pairs calculated by permutation test: 1283, 1548, End: endothelial cells: 1850, (treg T cells: 1128). Cells in mMφ, myCAF and End could significantly interact with cells in tT.

Since CS3 exhibited similarity with the immunosuppressive subtype S3 in TCGA, we analyzed the regulatory mechanisms and cell interactions that promote the formation of immune‐repressive T cells in this subtype. First, we obtained gene modules correlated with CS3‐specific subtypes (mT, tT, myCAF and mMφ). Thereafter, we combined WGCNA with TF results calculated using SCENIC to mine the key immunosuppression‐promoting TF regulons. In the WGCNA analysis, we treated the cell type information as a phenotype. We then obtained 12 gene modules from WGCNA (Fig. 6A), in which the green module was correlated with mT (*R* = 0.82, *P* < 0.001), magenta module with tT (*R* = 0.94, *P* < 0.001), yellow module with myCAF (*R* = 0.95, *P* < 0.001) and purple module with mMφ (*R* = 0.93, *P* < 0.001). Afterward, we obtained hub genes in each gene module (Section ‘Co‐expression network construction’). The mT hub genes were enriched in T cell selection and T cell differential pathways, whereas tT hub genes negatively regulated T cell activation and interleukin 10 secretion (Fig. S8A, Fig. 6B). Subsequently, we performed a hypergeometric test between the tT hub genes and TF regulon results and selected the critical TF (Section ‘SCENIC analysis’); the TF, *BATF*, was among the hits (Table S9). Of note, *BATF* could regulate *TIGIT* and *CTLA4* and the co‐stimulatory gene *ICOS* (Fig. 6C). The heatmap of TF‐activated scores in SCENIC also confirmed the cell‐type specificity of *BATF* in tT (Fig. S8B). We further confirmed the co‐expression of *BATF*, *CTLA4* and *TIGIT* in TCGA and ICGC cohorts. The results showed an obvious co‐occurrence between these gene pairs (Fig. 6D). High expression levels of *BATF* were correlated with poor prognosis in these two datasets (Fig. 6E, Fig. S8C). Critically, gene expression data from GSE149197 of *BATF*‐knockout Treg cells showed significantly lower *BATF, CTLA4, TIGIT* and *FOXP3* expression (Fig. 6F), and *BATF* was barely expressed in the healthy liver single‐cell dataset (GSE115469) (Fig. S8D). We then validated the function of *BATF* in two other single‐cell datasets (T cell dataset GSE98638 and TME dataset GSE146409) [9, 37]. The same data processing pipeline was used for these two datasets. There were nine T cell subtypes in GSE98638, including two immunosuppressive T‐reg subtypes (CD4‐CTLA4 and CD4‐FOXP3) and a T cell cluster in GSE146409 (Fig. S9A,B). In these two datasets, *BATF, TIGIT, LAG3* and *CTLA4* also had a co‐expression relationship (Fig. S9C,D). Moreover, *BATF* could regulate immunosuppressive genes in both datasets (Fig. S9E,F), further confirming its function. This indicates that TF *BATF* could play a critical role in forming immunosuppressive cells by upregulating the expression of immunosuppressive genes.

Because subtype S3 in TCGA had a high rate of stromal infiltration, we next analyzed the roles of the tumor microenvironment cells in the formation of a tumor immunosuppressive microenvironment. The cell interaction results showed that cells in tT could interact with CS3‐specific mMφ through chemokines *CXCL12_CXCR4*, *CCL4_CCR5* and *CCL3_CCR1*. Notably, mMφ was characterized by overexpression of the immune‐repressive gene *IL10* and could interact with tT via *NECTIN2_TIGIT*. tT could further suppress the immune response of T cells and ultimately promote the production of an immunosuppressive environment in HCC. In addition, mMφ frequently interacted with myCAF and endothelial cells in endothelial (End) type through chemokines such as *CXCL12_CXCR4* and growth factor *VEGFA_FLT1* (Fig. 6G). Meanwhile, endothelial cells in End could also interact with tT through *TIGIT_PVR*, which may also promote the formation of immune‐repressive cells. Therefore, mMφ could directly or indirectly promote the immunosuppressive status of the S3‐like HCC subtype.

## 4 Discussion

Immunotherapy is emerging as an important approach in cancer treatment, but its efficacy varies greatly among cancer patients. Due to its robust inflammatory pathogenesis, HCC remains a strong candidate for the development of immune‐based therapies. However, current immune checkpoint blockers have shown no benefits compared with sorafenib treatment, although the combination of atezolizumab and bevacizumab has shown promising effects in a front‐line phase III trial (IMbrave150). Systematic investigation of tumor‐infiltrating immune cells (TIICs) is critical for predicting the clinical outcomes and development of immunotherapies.

In the present study, we integrated multi‐single‐cell RNAseq and multi‐omics datasets to characterize the molecular heterogeneity of HCC at the inter‐ and intra‐tumor levels by analyzing the tumor‐infiltrating immune status in different patient groups. According to the infiltrating immune cell signatures, we identified three distinct subtypes: S1, S2 and S3. S1 and S3 had high rates of immune and stromal ratios, while S2 showed high tumor purity. S3, which had a high level of immunosuppressive signatures, was correlated with the poorest prognosis. Moreover, a set of signature genes was established, and multi bulk RNA and single‐cell datasets could be classified accurately using these genes. Our study also revealed an immunosuppression‐specific TF regulon and interaction network in the TME cells of subtype S3.

The location and characteristics of immune cells in the tumor microenvironment, as well as the response to immunotherapy (TME), are now recognized as prognostic [38]. In the present study, we obtained two high immune infiltration subtypes (S1 and S3) and one ‘cold tumor’ (S2). A previous study indicated that a high immune infiltration score was associated with a poor prognosis [39]. This subtype is similar to that of S3 in our data. However, we also obtained another immune subtype, which had a better prognosis with high levels of cytotoxic genes such as *PRF1* and *GZMA*, but low levels of *CTLA4* and *TIGIT*. These two heterogeneous immune subtypes were also present in multiple single‐cell datasets. These results indicate that patients with different immune characteristics have different prognoses. Moreover, there was no significant difference in TMB among the three subtypes, indicating the insufficiency of TMB in diagnosis for HCC immunotherapy. Recent results from randomized Phase III trials in front‐line non‐small cell lung cancer (NSCLC) also suggest that high TMB may not be effective at predicting survival benefits from a combination of *PD‐1* and *CTLA4* inhibitors [40]. Therefore, it is necessary to refer to both mutation and immune infiltration characteristics to evaluate immunotherapy efficacy.

Moreover, our study identified *BATF* as a critical modulator that upregulated *CTLA4, TIGIT* and *ICOS* expression. This observation was confirmed by the gene expression profiling of *BATF*‐knockout Treg cells. *BATF* is a basic leucine zipper (bZIP) TF required to produce *IL17*, *IL21* and *IL23* receptors in TH17 cells. In TH17 cells, *BATF* is thought to function as a ‘pioneer factor’, together with *IRF4*, mediating chromatin remodeling [41]. Transcriptional analysis of HIV‐specific CD8^+^ T cells showed that PD‐1 inhibits T cell function by upregulating *BATF* [42]. Our data showed for the first time that *BATF* could promote the formation of immunosuppressive T cells to inhibit the immune response in HCC. These results indicate that *BATF* inhibitors might change the immunosuppressed tumor to an immune status with a better prognosis, which might facilitate HCC immunotherapy.

Tumor microenvironment is permissive of existing functional T cell responses [40]. In the current study, there was a high rate of macrophages overexpressing *CD68* in the S3 subtype in bulk RNAseq data. Similar characteristics were observed in the S3‐like single‐cell samples. It has been demonstrated that TAMs overexpressing *SLC40A1* and *GPNMB* are associated with poor prognosis [36]. However, we found that the MDSC‐like macrophage mMφ also plays a vital role in formation of immunosuppressive T cells. mMφ co‐existed with M1 and M2 signatures and showed a high level of *IL10*. It can interact with fibroblasts and endothelial cells via chemokines and growth factors. The interaction network likely promotes the immunosuppressed state in the TME of HCC.

## 5 Conclusions

This study investigated the inter‐ and intra‐tumor heterogeneity of HCC using both bulk and single‐cell transcriptomic data. Three three distinct subtypes, S1, S2 and S3, were identified. In these subtypes, S3, with the poorest prognosis, had a high degree of macrophage and immunosuppressive T cell infiltration. Our study suggests that the TF *BATF* in Treg cells well as MDSC‐like macrophage cells could promote the formation of immunosuppressive cells and affect the prognosis of the HCC patients. These discoveries could facilitate clinical diagnosis and treatment of HCC.

## Conflict of interest

The authors declare no conflict of interest.

## Author contributions

KY, CX and XY developed the study concept. TW directed experimental design and interpreted data. TW performed computational analysis. TW wrote the manuscript. ND performed the collection of TCGA and ICGC gene expression data. GT, ZL and XL collected and interpreted *BATF* bulk RNA data. ZX, BS and LL interpreted the clinical impact of this study. All authors read, edited and approved the manuscript.

### Peer Review

The peer review history for this article is available at https://publons.com/publon/10.1002/1878‐0261.13190.

## Supporting information


**Fig. S1.** NMF clustering related results and in TCGA and ICGC cohorts. (A) Cophenetic coefficient result of NMF clustering in TCGA cohort. The intensely dropped of cophenetic coefficient values at cluster number 3 indicate the appropriate cluster number. (B) Heatmap showing the consensus result in ICGC cohort. (C) Cophenetic coefficient result of NMF in ICGC cohort. The intensely dropped of cophenetic coefficient values at cluster number 3 indicating the appropriate cluster number. (D) Overall survival curves showing the prognosis result among the three subtypes (S1, S2 and S3) in the ICGC cohort. Statistical significance was calculated using the log‐rank test (S1:72, S2:44, S3:116). (E) Heatmap showing the consistency analysis result among the subtypes in the TCGA and ICGC cohort, in which red indicates *P* < 0.001 and blue *P* > 0.05.

**Fig. S2.** Inter‐tumor heterogeneity of immunology and mutation correlation in the three HCC subtypes. (A) Boxplots showing the stromal score (S1:120, S2:144, S3:89; nonsignificant (ns) *P* > 0.05, *P < 0.05, **P < 0.01, ***P < 0.001). Pairwise comparison was conducted by Wilcoxon rank‐sum test in the TCGA LIHC cohort. In the boxplot, the centerline represents the median and box limits represent upper and lower quartiles. Each dot represents a sample. (B) Boxplots showing the immunosuppressed score in distinct three malignant subtypes (S1:120, S2:144, S3:89. ns *P* > 0.05, *P < 0.05, **P < 0.01, ***P < 0.001). Pairwise comparison was conducted by Wilcoxon rank‐sum test in the TCGA LIHC cohort. In the boxplot, the centerline represents the median and box limits represent upper and lower quartiles. Each dot represents a sample. (C) Boxplots showing the expression of aT in distinct three malignant subtypes (S1:120, S2:144, S3:89. ns *P* > 0.05, *P < 0.05, **P < 0.01, ***P < 0.001). Pairwise comparison was carried out by Wilcoxon rank‐sum test in the TCGA LIHC cohort. In the boxplot, the centerline represents the median and box limits represent upper and lower quartiles. Each dot represents a sample. (D) Boxplots showing the expression of immune genes in distinct three malignant subtypes (S1:120, S2:144, S3:89; ns *P* > 0.05, *P < 0.05, **P < 0.01, ***P < 0.001). Pairwise comparison was carried out by Wilcoxon rank‐sum test in the TCGA LIHC cohort. In the boxplot, the centerline represents the median and box limits represent upper and lower quartiles. Each dot represents a sample. (E) Boxplots showing the expression of macrophage and EMT genes in distinct three malignant subtypes (S1:120, S2:144, S3:89; ns *P* > 0.05, *P < 0.05, **P < 0.01, ***P < 0.001). Pairwise comparison was conducted by Wilcoxon rank‐sum test in the TCGA LIHC cohort. In the boxplot, the centerline represents the median and box limits represent upper and lower quartiles. Each dot represents a sample. (F) Correlation heatmap showing the co‐occurrence and mutually exclusive occurrence of the mutation genes. Color key from light to dark indicates significant *P*‐value from low to high (/ P < 0.05, *P* < 0.1, Pearson's correlation test in the TCGA LIHC cohort).

**Fig. S3.** Profile of mutation‐related genes in the different subtypes. (A) Boxplots showing the expression of *CTNNB1* and *MYC* in the three subtypes (S1:120, S2:144, S3:89; ns *P* > 0.05, *P < 0.05, **P < 0.01, ***P < 0.001). Pairwise comparison was conducted by Wilcoxon rank‐sum test in the TCGA LIHC cohort. In the boxplot, the centerline represents the median and box limits represent upper and lower quartiles. Each dot represents a sample. (B) Boxplots showing the immune score and tumor purity in the CTNNB1‐mutation and CTNNB1‐nonMutation groups (*CTNNB1*‐mut: 90; *CTNNB1*‐nonmut: 263; ns *P* > 0.05, *P < 0.05, **P < 0.01, ***P < 0.001). Pairwise comparison was conducted by Wilcoxon rank‐sum test in the TCGA LIHC cohort. In the boxplot, the centerline represents the median and box limits represent upper and lower quartiles. Each dot represents a sample. (C) Boxplots showing the expression of *CD3D* and *CTLA4* in the CTNNB1‐mutation and CTNNB1‐nonMutation groups (CTNNB1‐mut: 90; CTNNB1‐nonmut: 263; ns *P* > 0.05, *P < 0.05, **P < 0.01, ***P < 0.001). Pairwise comparison was conducted by Wilcoxon rank‐sum test in the TCGA LIHC cohort. In the boxplot, the centerline represents the median and box limits represent upper and lower quartiles. Each dot represents a sample.

**Fig. S4.** Inter‐tumor heterogeneity of CNV mutation profile in the three HCC subtypes. (A) Boxplots showing the expression of *CNV*‐related genes in the three subtypes (S1:120, S2:144, S3:89;. ns *P* > 0.05, *P < 0.05, **P < 0.01, ***P < 0.001). Pairwise comparison was conducted by Wilcoxon rank‐sum test in the TCGA LIHC cohort. In the boxplot, the centerline represents the median and box limits represent upper and lower quartiles. Each dot represents a sample. (B) Amplification regions in the three subtypes: columns represent the chromosomal regions and rows represent significance of enrichment calculated by GISTIC2. (C) Deletion regions in the three subtypes: columns represent the chromosomal regions and rows represent significance of enrichment calculated by GISTIC2.

**Fig. S5.** Validation of the classifier and comparison of the immune profile of the subtypes between ICGC and TCGA using the classifier. (A) Overall survival curves showing the prognosis results for the three subtypes (S1, S2 and S3) obtained from NMF clustering using the 108 genes in the TCGA LIHC cohort. Statistical significance was calculated using the log‐rank test (S1:149, S2:92, S3:112 in the TCGA LIHC cohort). (B) Overall survival curves showing the prognosis result for the three subtypes (S1, S2 and S3) in the ICGC cohort obtained from NMF clustering using the 108 genes. Statistical significance was calculated using the log‐rank test (S1:93, S2:98, S3:41). (C) Boxplots show the expression of immune genes in ICGC cohort (S1:93, S2:98, S3:41; ns *P* > 0.05, *P < 0.05, **P < 0.01, ***P < 0.001, Wilcoxon rank‐sum test). In the boxplot, the centerline represents the median and box limits represent upper and lower quartiles. Each dot represents a sample. (D) Heatmap showing the consistency analysis result among the subtypes in the TCGA and ICGC cohort, in which red indicates *P* < 0.001 and blue *P* > 0.05. (E) Boxplots showing the tumor purity scores in the three subtypes in the ICGC cohort (S1:93, S2:98, S3:41; ns *P* > 0.05, *P < 0.05, **P < 0.01, ***P < 0.001, Wilcoxon rank‐sum test). In the boxplot, the centerline represents the median and box limits represent upper and lower quartiles. Each dot represents a sample. (F) Boxplots showing immune scores of the three subtypes in the ICGC cohort (S1:93, S2:98, S3:41; ns *P* > 0.05, *P < 0.05, **P < 0.01, ***P < 0.001, Wilcoxon rank‐sum test). In the boxplot, the centerline represents the median and box limits represent upper and lower quartiles. Each dot represents a sample.

**Fig. S6.** Consistency of pathways between the two cohorts. (A) Dot plot showing the enrichment pathways in TCGA. Dot size showing the enrichment score and color from blue to red indicates the −log10 (*P*‐value) of the enrichment pathways. (B) Dot plot showing the enrichment pathways in ICGC. Dot size showing the enrichment score and color from blue to red indicate the −log_10_ (*P*‐value) of the pathways in the enrichment.

**Fig. S7.** Expression of marker genes in GSE149614. (A) Violin plots showing the expression profile of marker genes in distinct subtypes (mMφ: 1283, myCAF: 1548, End: 1850, tT: 1128, aNK: 350, mT: 2763, tT: 1128, pT: 516, aT: 1600, nB: 1436, mB: 409, H1: 9855, H2: 1143, H3: 821, H4: 2733). In the violin plot, the centerline represents the median and box limits represent upper and lower quartiles; whiskers, data range. (B) Immune gene expression profile in the single‐cell RNAseq cohort GSE149614, colored from gray to red indicating the expression level from low to high. (C) Stromal gene expression profiles in the single‐cell RNAseq cohort GSE149614, colored from gray to red, indicating the expression level from low to high.

**Fig. S8.** Gene enrichment and validation of the function of BATF in GSE149614. (A) Gene enrichment result in mT subtype (mT). (B) Heatmap showing the specificity of TF activation scores in the four T cell subtypes calculated by SCENIC. Color from blue to red indicates TF activation scores from low to high. (C) Overall survival curves showing the prognosis result of TF *BATF* in the ICGC cohort. Red and blue color indicates patients with a high expression level of *BATF* and low level in this cohort. The grouping cutoff value was calculated by X‐tile. Statistical significance was calculated using the log‐rank test (BATF‐High: 59, BATF‐Low: 173). (D) Expression of critical TF *BATF* in healthy human sample. Color key from white to red indicates the gene expression level from low to high.

**Fig. S9.** Expression of *BATF* and regulon in the other two single‐cell datasets. (A) The UMAP showing the profile of 4934 cells from single‐cell RNAseq dataset GSE98638. Clusters are indicated by different colors. (B) The UMAP showing the profile of 7947 cells from single‐cell RNAseq dataset GSE146409. Clusters are indicated by different colors. cDC: classical DC; SAM: scar‐associated macrophages; TM1: tissue monocytes; CAF: cancer‐associated fibroblasts; LVEC: liver vascular endothelial cells; LESC: liver sinusoidal endothelial cells; vSMC: vascular smooth muscle cells. (C) Gene expression pattern of immune genes in the GSE98638. Color from white to red indicates the gene expression level from low to high. (D) Gene expression pattern of immune genes in GSE146409 dataset. (E) BATF‐regulon of immunosuppressive T cells in GSE98638, in which red node indicates TF and yellow ones indicate target genes. (F) BATF‐regulon of immunosuppressive T cells in GSE146409, in which red node indicates TF and yellow ones indicate target genes.

**Table S1.** Datasets applied in the study.
**Table S2.** Clinical characteristics among the three tumor subtypes of LIHC in TCGA cohort. LIHC, liver hepatocellular carcinoma.
**Table S3.** GSVA result showing the inter‐tumor heterogeneity of enriched pathways among the three subtypes in TCGA cohort.
**Table S4.** Mutation characteristics in distinct HCC classification.
**Table S5.** FRP and TPR result to identify the ‘hot tumor’ samples.
**Table S6.** FRP and TPR result to identify the ‘cold tumor’ samples.
**Table S7.** Classifier signatures, repressed and liver marker genes.
**Table S8.** Cell types and marker genes using in the cluster definition.
**Table S9.** mMφ‐related TF result obtained from SCENIC.

## Data Availability

All data in our study are available upon request.
