# Supplementary figures and images for: Integrating bulk and single‐cell RNA sequencing reveals cellular heterogeneity and immune infiltration in hepatocellular carcinoma
Source: Mol Oncol. 2022 Mar 1;16(11):2195–213. doi: 10.1002/1878-0261.13190 (PMC9168757; doi:10.1002/1878-0261.13190)

Figure S1

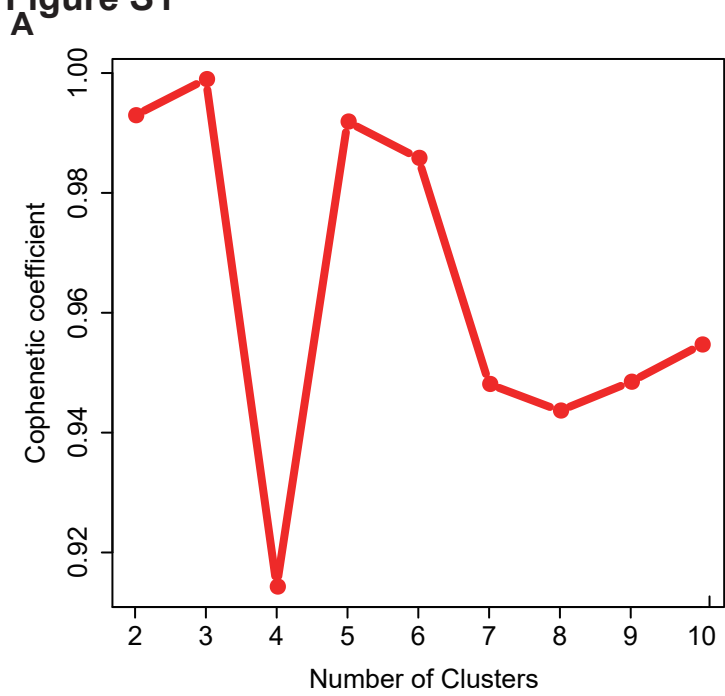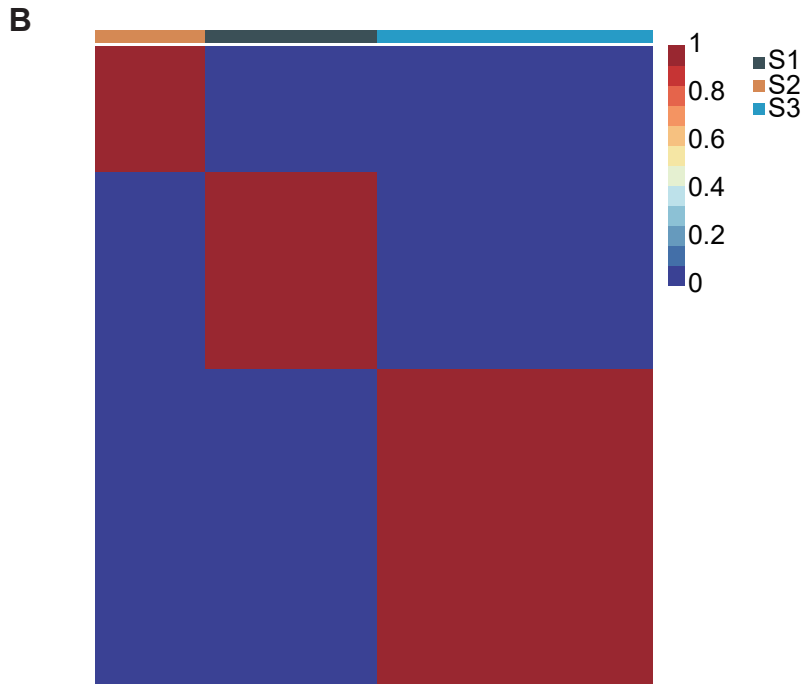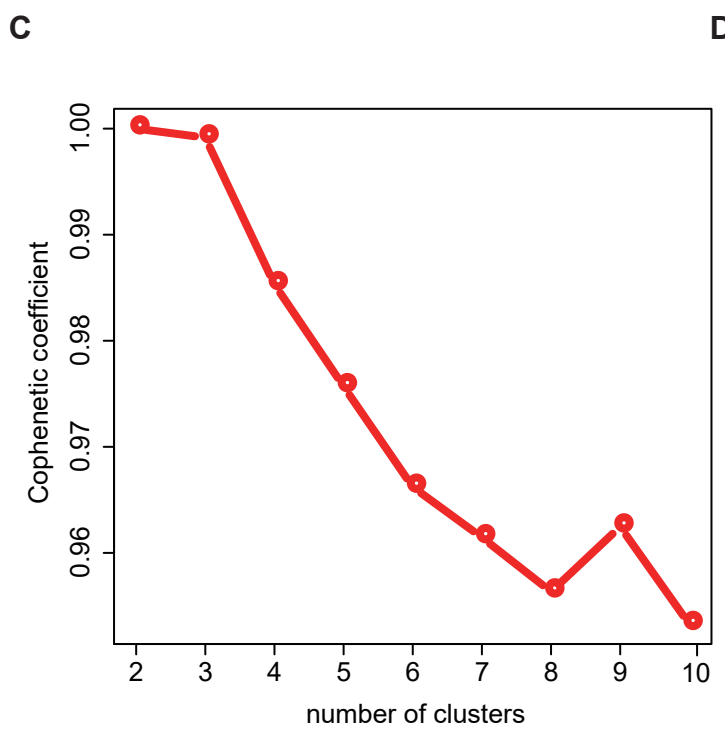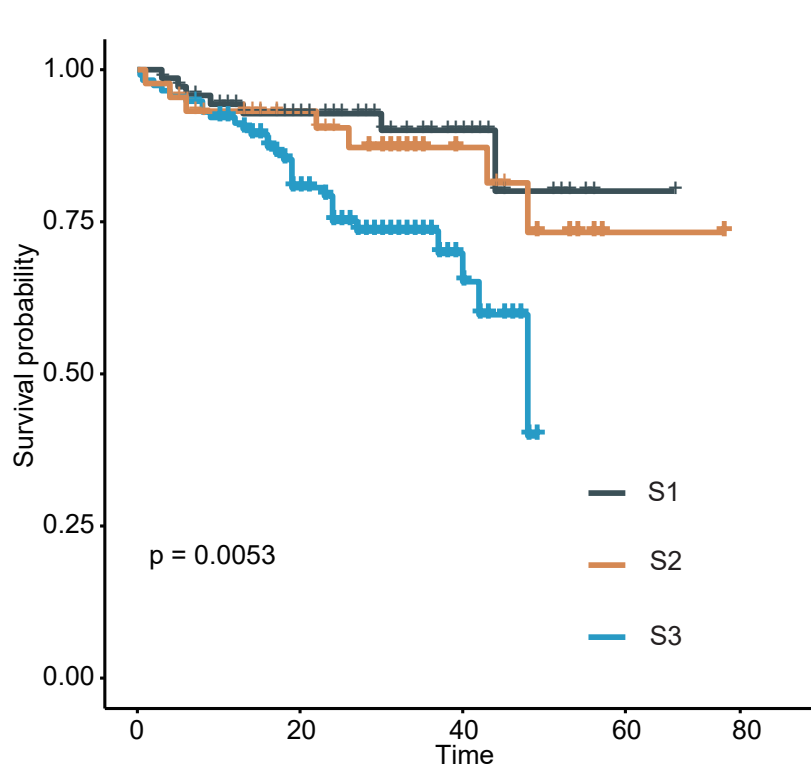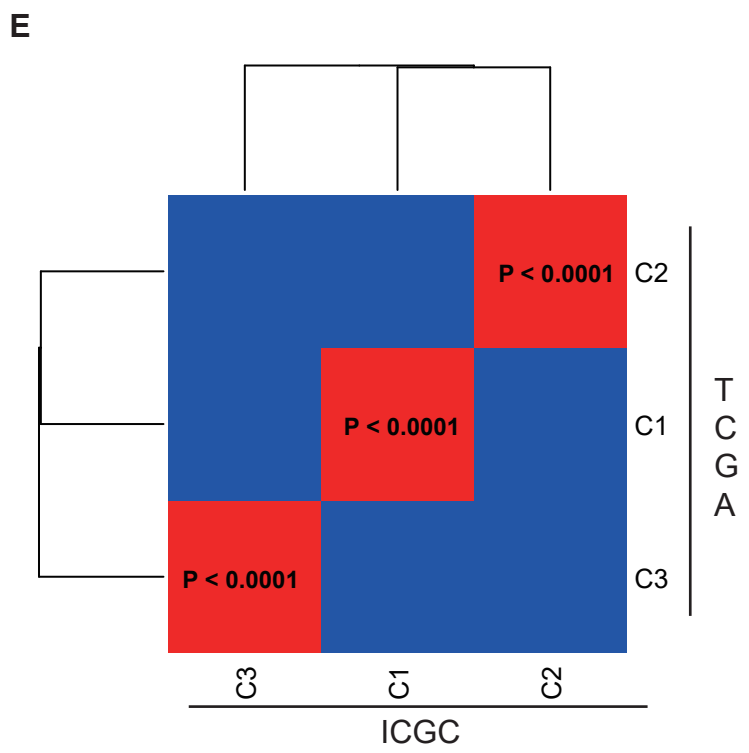

Supplement: Supplementary file 1 — Fig. S1. NMF clustering related results and in TCGA and ICGC cohorts. (A) Cophenetic coefficient result of NMF clustering in TCGA cohort. The intensely dropped of cophenetic coefficient values at cluster number 3 indicate the appropriate cluster number. (B) Heatmap showing the consensus result in ICGC cohort. (C) Cophenetic coefficient result of NMF in ICGC cohort. The intensely dropped of cophenetic coefficient values at cluster number 3 indicating the appropriate cluster number. (D) Overall survival curves showing the prognosis result among the three subtypes (S1, S2 and S3) in the ICGC cohort. Statistical significance was calculated using the log‐rank test (S1:72, S2:44, S3:116). (E) Heatmap showing the consistency analysis result among the subtypes in the TCGA and ICGC cohort, in which red indicates P < 0.001 and blue P > 0.05. [file MOL2-16-2195-s009.pdf]

**Figure S2****A**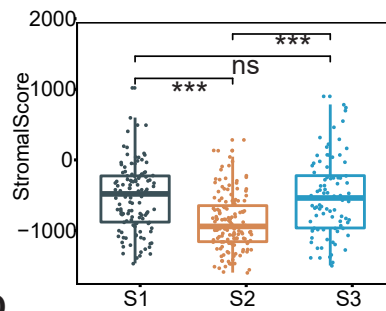**B**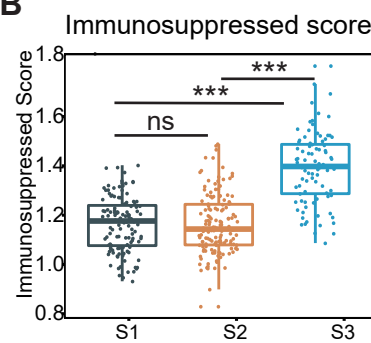**C**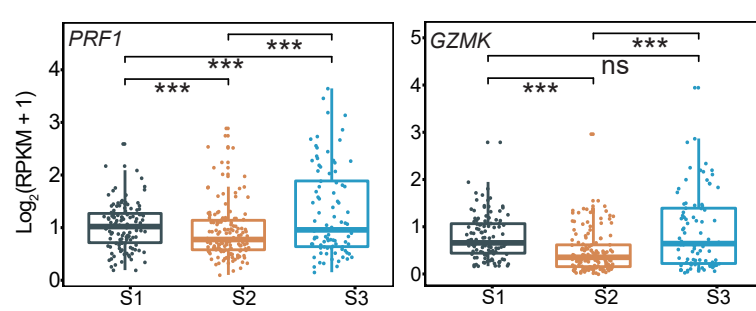**D**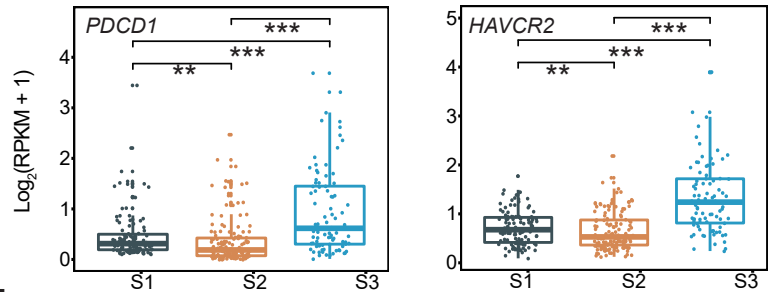**E**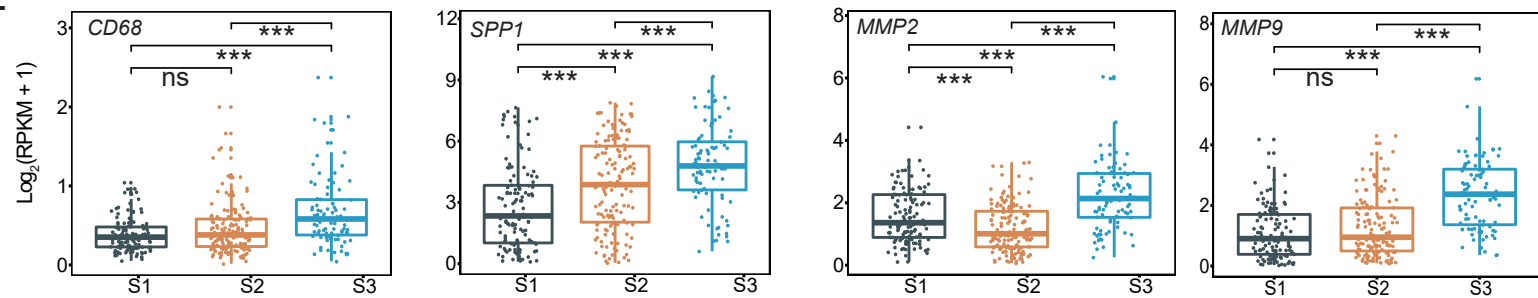**F**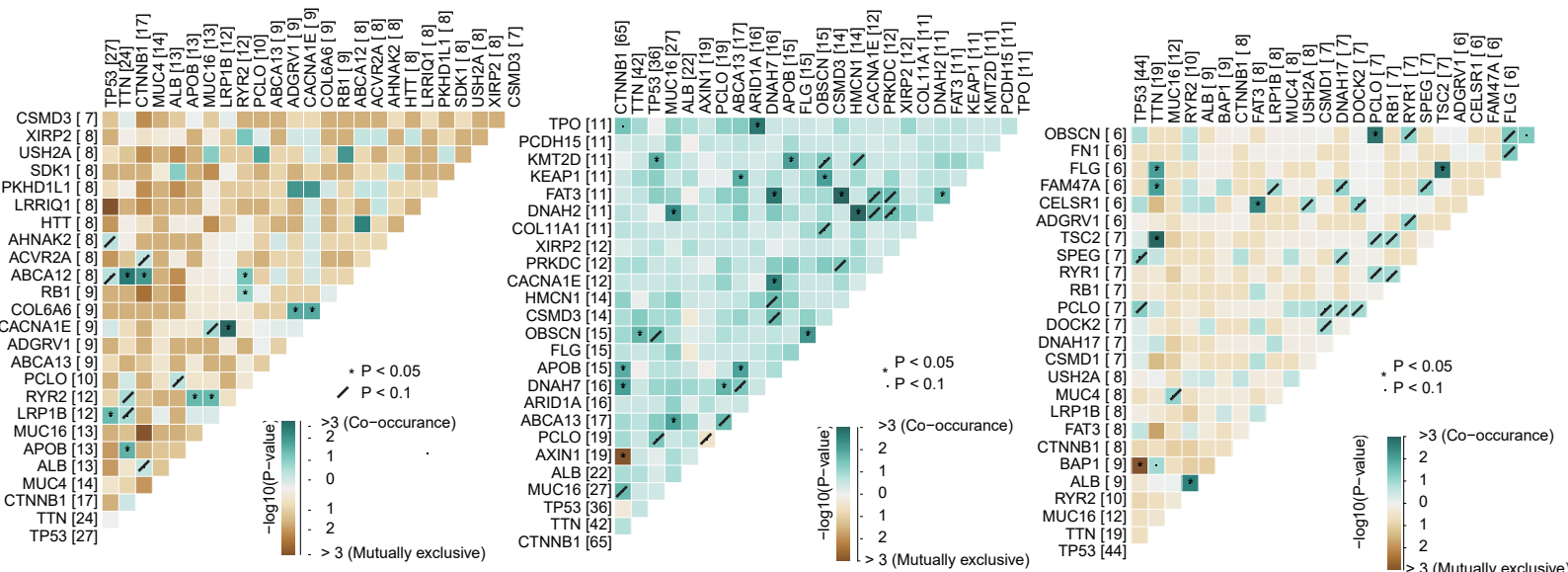

Supplement: Supplementary file 2 — Fig. S2. Inter‐tumor heterogeneity of immunology and mutation correlation in the three HCC subtypes. (A) Boxplots showing the stromal score (S1:120, S2:144, S3:89; nonsignificant (ns) P > 0.05, *P < 0.05, **P < 0.01, ***P < 0.001). Pairwise comparison was conducted by Wilcoxon rank‐sum test in the TCGA LIHC cohort. In the boxplot, the centerline represents the median and box limits represent upper and lower quartiles. Each dot represents a sample. (B) Boxplots showing the immunosuppressed score in distinct three malignant subtypes (S1:120, S2:144, S3:89. ns P > 0.05, *P < 0.05, **P < 0.01, ***P < 0.001). Pairwise comparison was conducted by Wilcoxon rank‐sum test in the TCGA LIHC cohort. In the boxplot, the centerline represents the median and box limits represent upper and lower quartiles. Each dot represents a sample. (C) Boxplots showing the expression of aT in distinct three malignant subtypes (S1:120, S2:144, S3:89. ns P > 0.05, *P < 0.05, **P < 0.01, ***P < 0.001). Pairwise comparison was carried out by Wilcoxon rank‐sum test in the TCGA LIHC cohort. In the boxplot, the centerline represents the median and box limits represent upper and lower quartiles. Each dot represents a sample. (D) Boxplots showing the expression of immune genes in distinct three malignant subtypes (S1:120, S2:144, S3:89; ns P > 0.05, *P < 0.05, **P < 0.01, ***P < 0.001). Pairwise comparison was carried out by Wilcoxon rank‐sum test in the TCGA LIHC cohort. In the boxplot, the centerline represents the median and box limits represent upper and lower quartiles. Each dot represents a sample. (E) Boxplots showing the expression of macrophage and EMT genes in distinct three malignant subtypes (S1:120, S2:144, S3:89; ns P > 0.05, *P < 0.05, **P < 0.01, ***P < 0.001). Pairwise comparison was conducted by Wilcoxon rank‐sum test in the TCGA LIHC cohort. In the boxplot, the centerline represents the median and box limits represent upper and lower quartiles. Each dot represents a sample. (F) Correl [file MOL2-16-2195-s002.pdf]

**Figure S3**

**A**

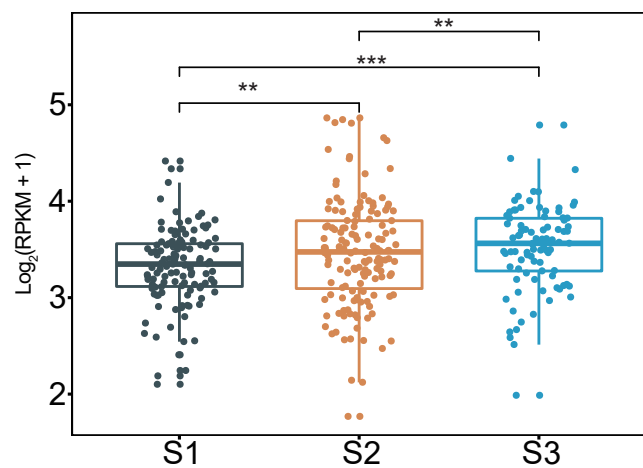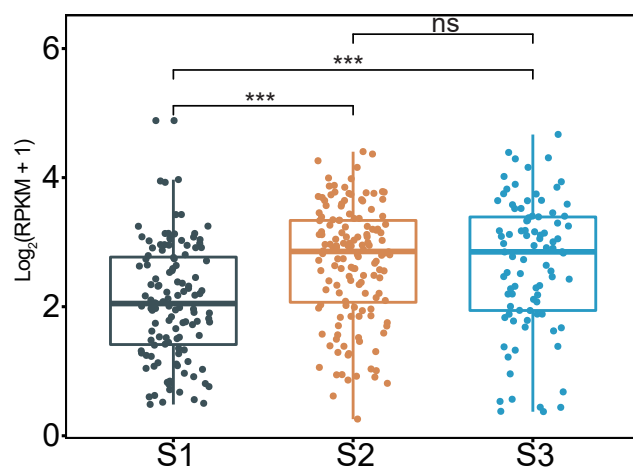

**B**

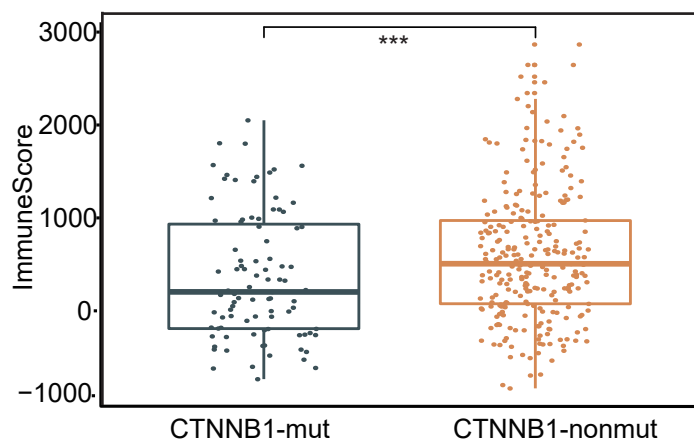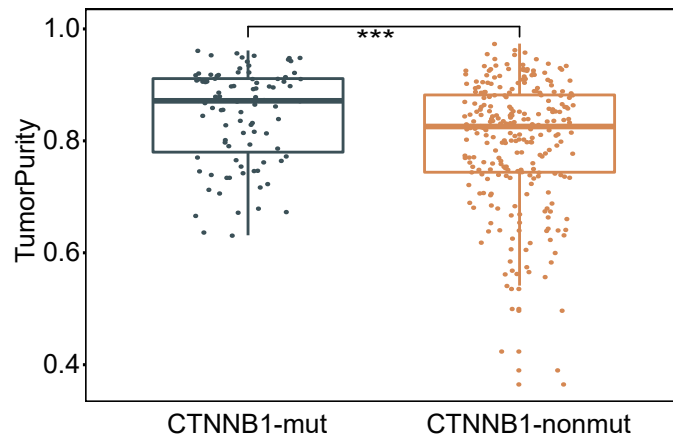

**C**

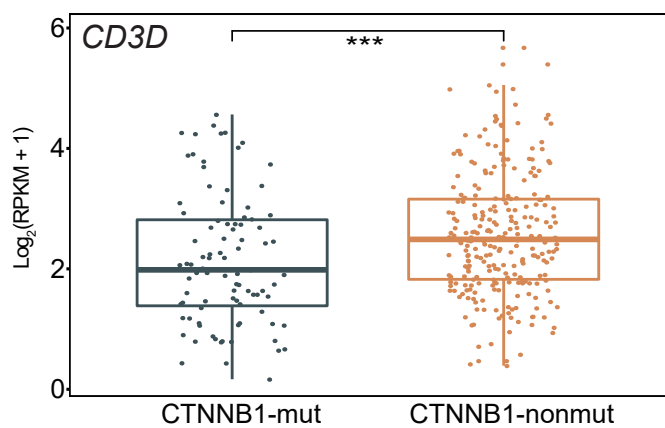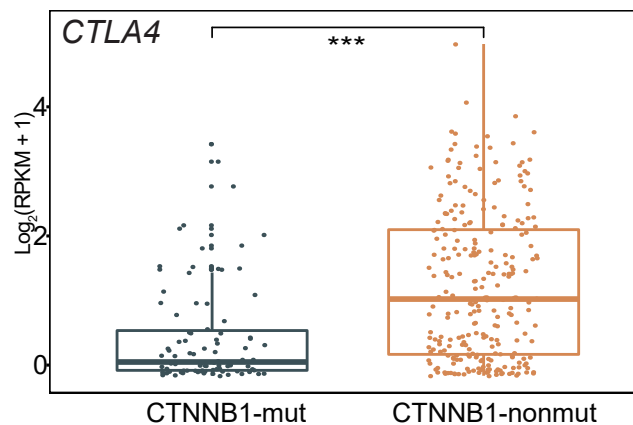

Supplement: Supplementary file 3 — Fig. S3. Profile of mutation‐related genes in the different subtypes. (A) Boxplots showing the expression of CTNNB1 and MYC in the three subtypes (S1:120, S2:144, S3:89; ns P > 0.05, *P < 0.05, **P < 0.01, ***P < 0.001). Pairwise comparison was conducted by Wilcoxon rank‐sum test in the TCGA LIHC cohort. In the boxplot, the centerline represents the median and box limits represent upper and lower quartiles. Each dot represents a sample. (B) Boxplots showing the immune score and tumor purity in the CTNNB1‐mutation and CTNNB1‐nonMutation groups (CTNNB1‐mut: 90; CTNNB1‐nonmut: 263; ns P > 0.05, *P < 0.05, **P < 0.01, ***P < 0.001). Pairwise comparison was conducted by Wilcoxon rank‐sum test in the TCGA LIHC cohort. In the boxplot, the centerline represents the median and box limits represent upper and lower quartiles. Each dot represents a sample. (C) Boxplots showing the expression of CD3D and CTLA4 in the CTNNB1‐mutation and CTNNB1‐nonMutation groups (CTNNB1‐mut: 90; CTNNB1‐nonmut: 263; ns P > 0.05, *P < 0.05, **P < 0.01, ***P < 0.001). Pairwise comparison was conducted by Wilcoxon rank‐sum test in the TCGA LIHC cohort. In the boxplot, the centerline represents the median and box limits represent upper and lower quartiles. Each dot represents a sample. [file MOL2-16-2195-s010.pdf]

**Figure S4**  
**A**

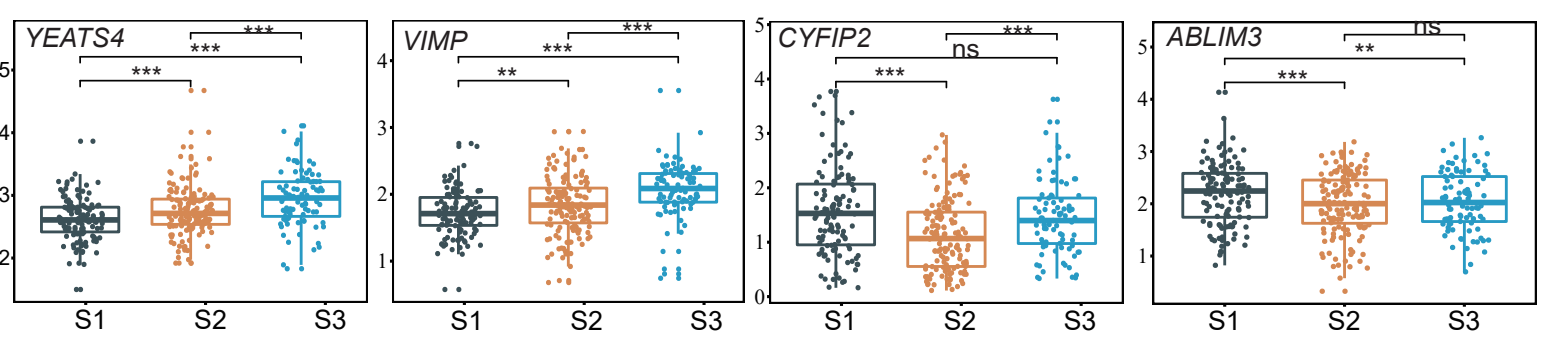

**B**

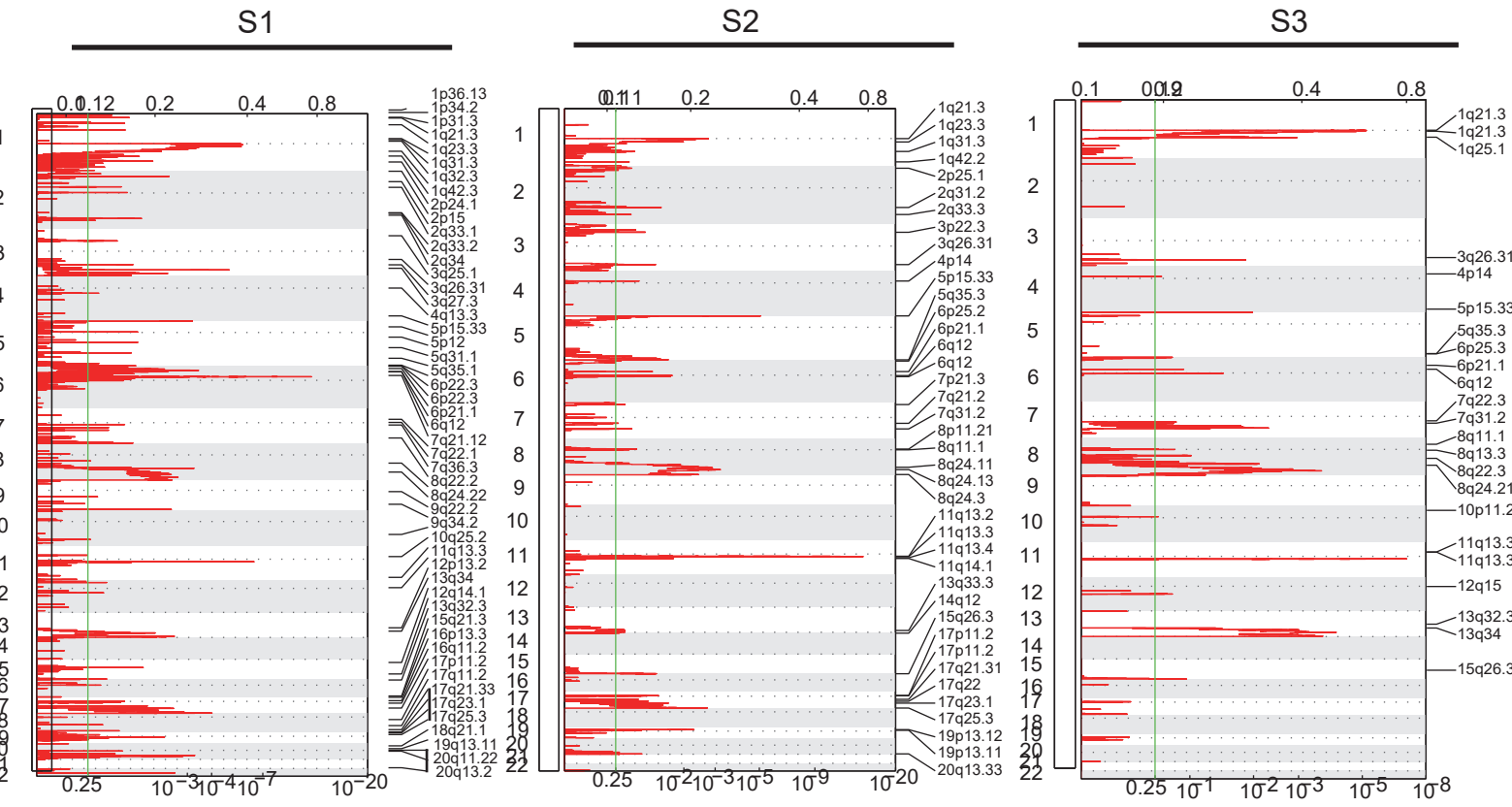

**C**

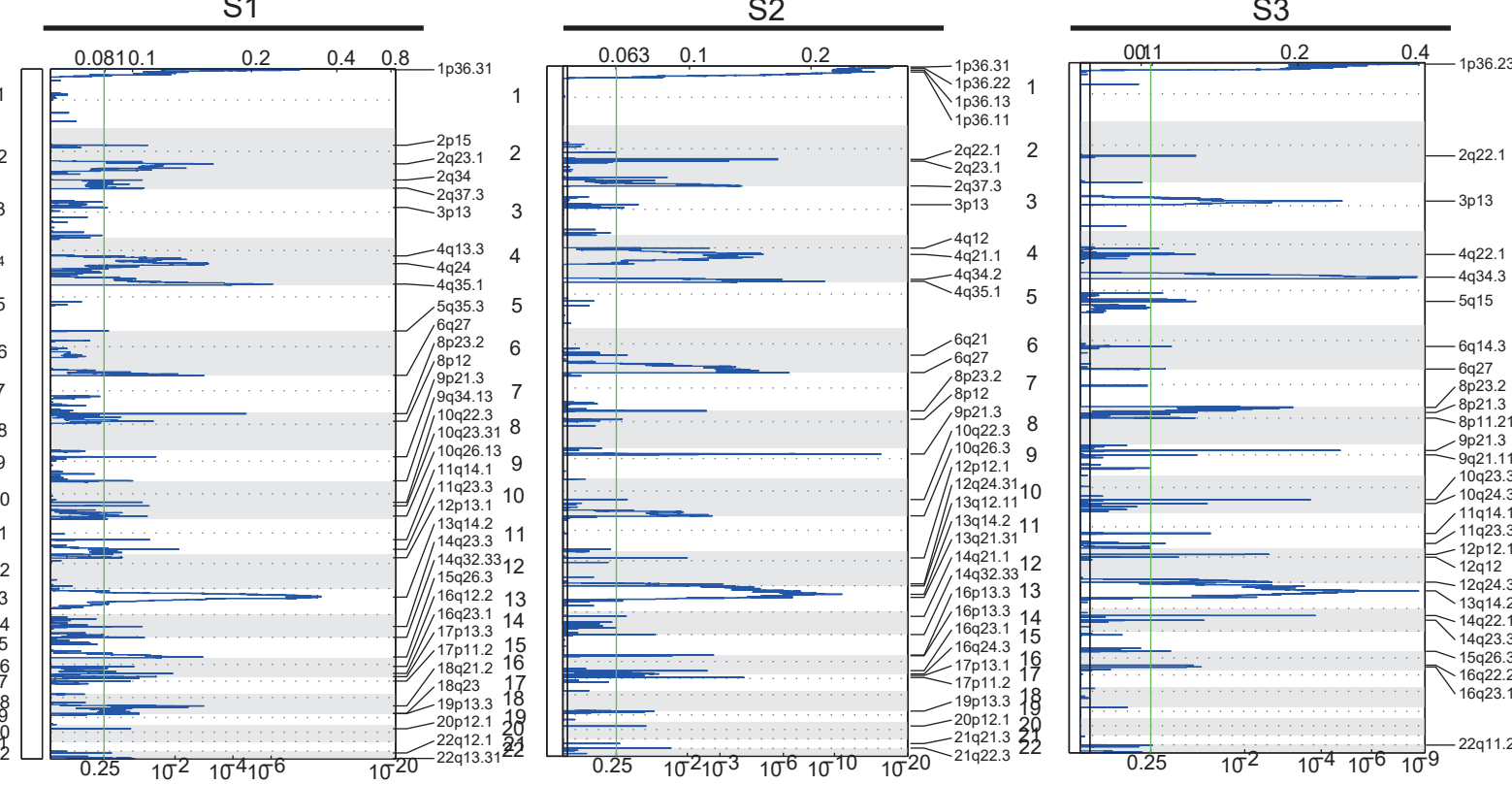

Supplement: Supplementary file 4 — Fig. S4. Inter‐tumor heterogeneity of CNV mutation profile in the three HCC subtypes. (A) Boxplots showing the expression of CNV‐related genes in the three subtypes (S1:120, S2:144, S3:89;. ns P > 0.05, *P < 0.05, **P < 0.01, ***P < 0.001). Pairwise comparison was conducted by Wilcoxon rank‐sum test in the TCGA LIHC cohort. In the boxplot, the centerline represents the median and box limits represent upper and lower quartiles. Each dot represents a sample. (B) Amplification regions in the three subtypes: columns represent the chromosomal regions and rows represent significance of enrichment calculated by GISTIC2. (C) Deletion regions in the three subtypes: columns represent the chromosomal regions and rows represent significance of enrichment calculated by GISTIC2. [file MOL2-16-2195-s005.pdf]

**Figure S5****A**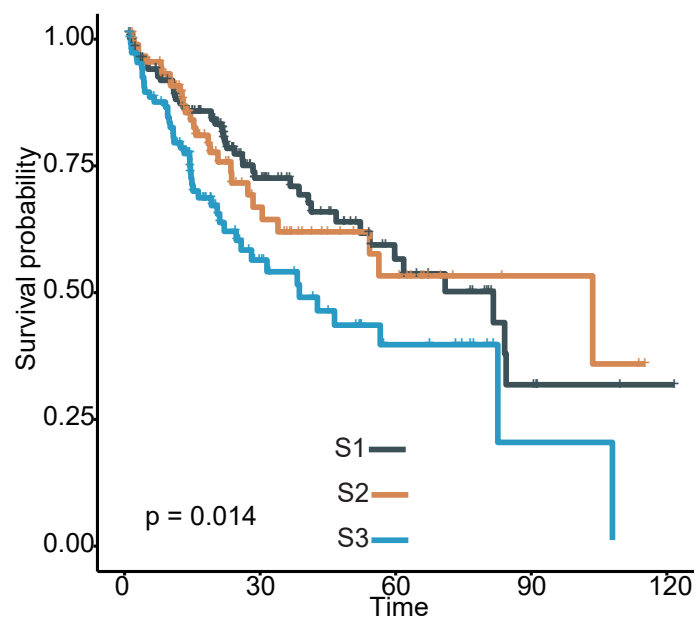**B**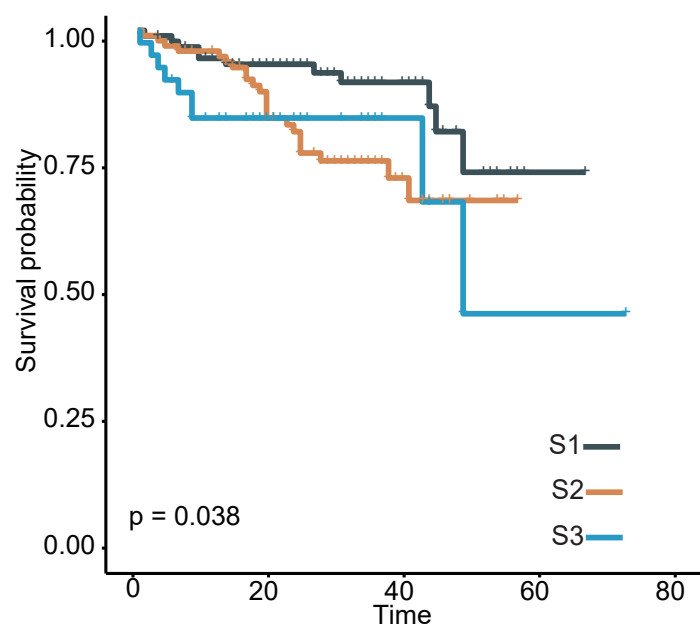**C**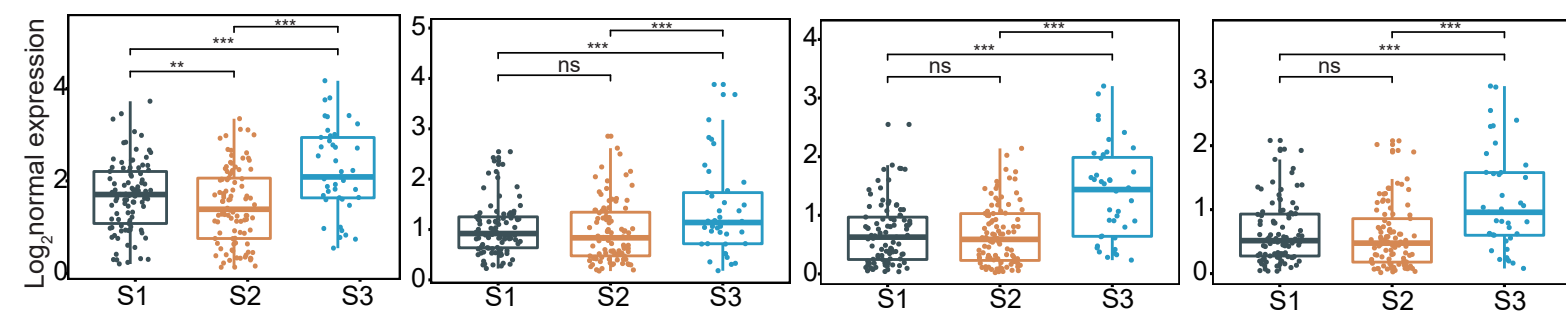**D**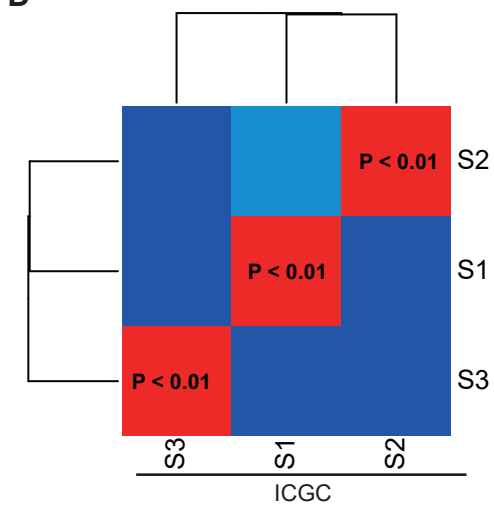**E**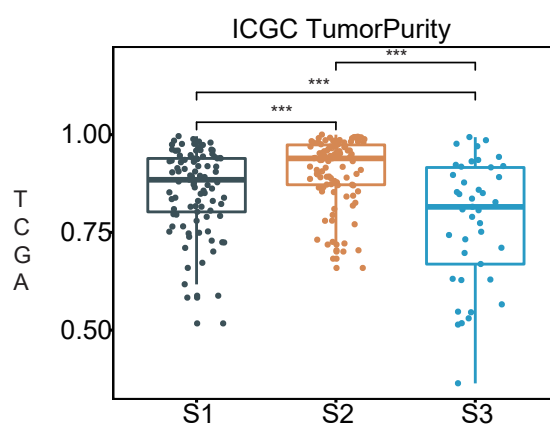**F**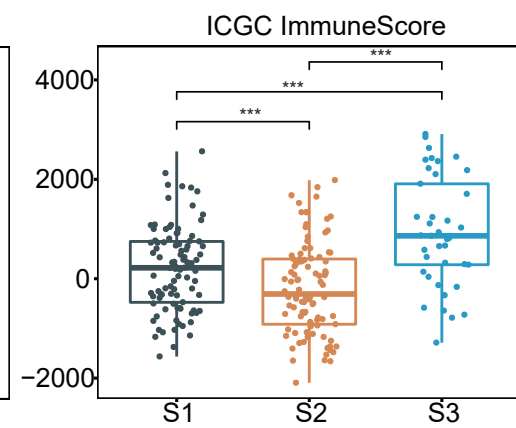

Supplement: Supplementary file 5 — Fig. S5. Validation of the classifier and comparison of the immune profile of the subtypes between ICGC and TCGA using the classifier. (A) Overall survival curves showing the prognosis results for the three subtypes (S1, S2 and S3) obtained from NMF clustering using the 108 genes in the TCGA LIHC cohort. Statistical significance was calculated using the log‐rank test (S1:149, S2:92, S3:112 in the TCGA LIHC cohort). (B) Overall survival curves showing the prognosis result for the three subtypes (S1, S2 and S3) in the ICGC cohort obtained from NMF clustering using the 108 genes. Statistical significance was calculated using the log‐rank test (S1:93, S2:98, S3:41). (C) Boxplots show the expression of immune genes in ICGC cohort (S1:93, S2:98, S3:41; ns P > 0.05, *P < 0.05, **P < 0.01, ***P < 0.001, Wilcoxon rank‐sum test). In the boxplot, the centerline represents the median and box limits represent upper and lower quartiles. Each dot represents a sample. (D) Heatmap showing the consistency analysis result among the subtypes in the TCGA and ICGC cohort, in which red indicates P < 0.001 and blue P > 0.05. (E) Boxplots showing the tumor purity scores in the three subtypes in the ICGC cohort (S1:93, S2:98, S3:41; ns P > 0.05, *P < 0.05, **P < 0.01, ***P < 0.001, Wilcoxon rank‐sum test). In the boxplot, the centerline represents the median and box limits represent upper and lower quartiles. Each dot represents a sample. (F) Boxplots showing immune scores of the three subtypes in the ICGC cohort (S1:93, S2:98, S3:41; ns P > 0.05, *P < 0.05, **P < 0.01, ***P < 0.001, Wilcoxon rank‐sum test). In the boxplot, the centerline represents the median and box limits represent upper and lower quartiles. Each dot represents a sample. [file MOL2-16-2195-s006.pdf]

Figure S6

A

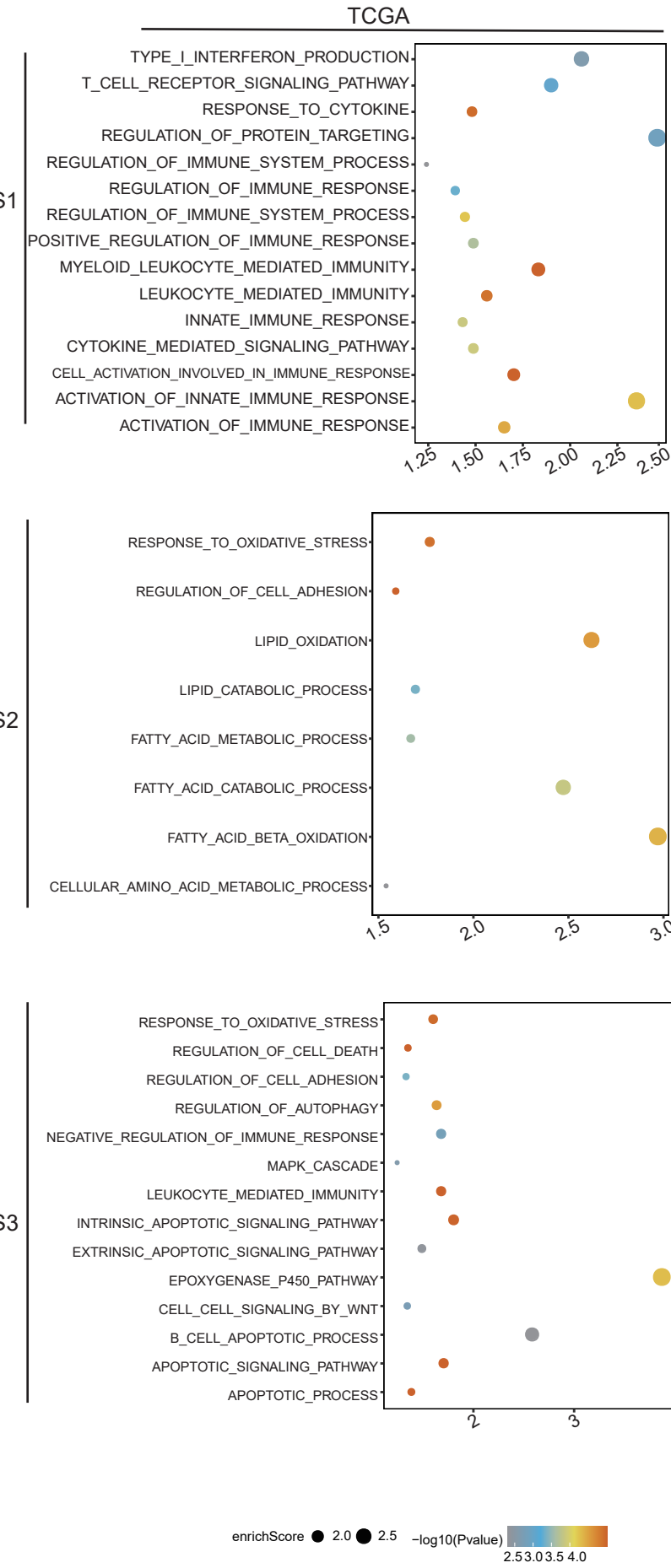

B

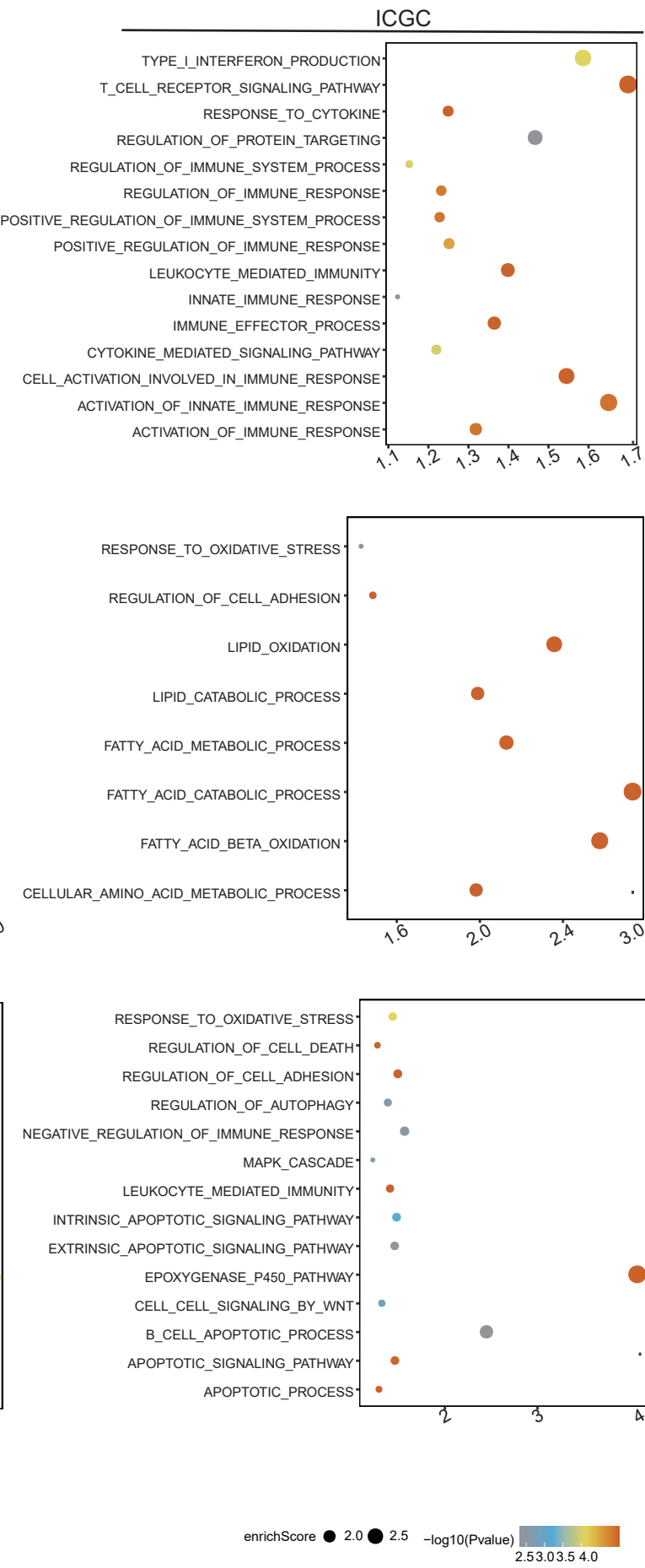

Supplement: Supplementary file 6 — Fig. S6. Consistency of pathways between the two cohorts. (A) Dot plot showing the enrichment pathways in TCGA. Dot size showing the enrichment score and color from blue to red indicates the −log10 (P‐value) of the enrichment pathways. (B) Dot plot showing the enrichment pathways in ICGC. Dot size showing the enrichment score and color from blue to red indicate the −log10 (P‐value) of the pathways in the enrichment. [file MOL2-16-2195-s003.pdf]

Figure S7

A

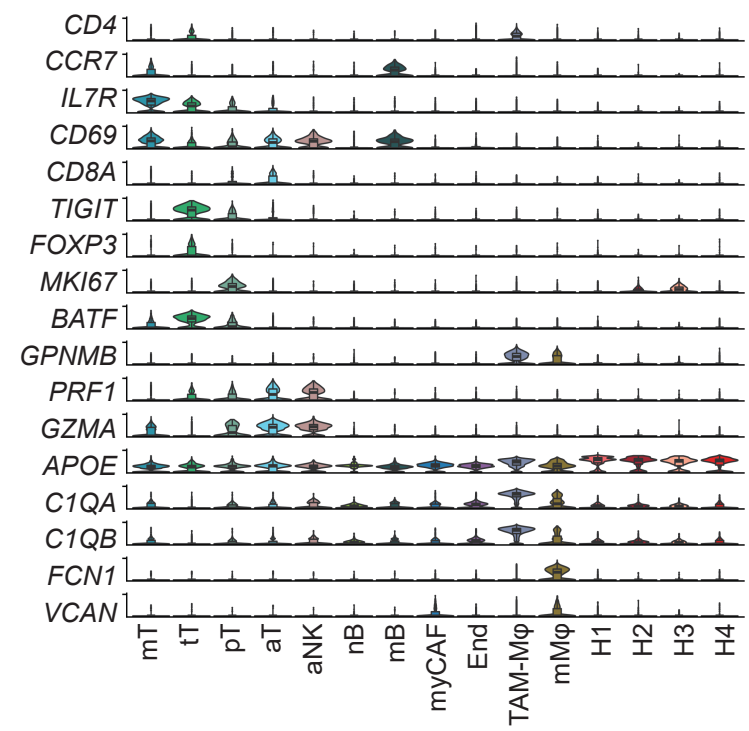

B

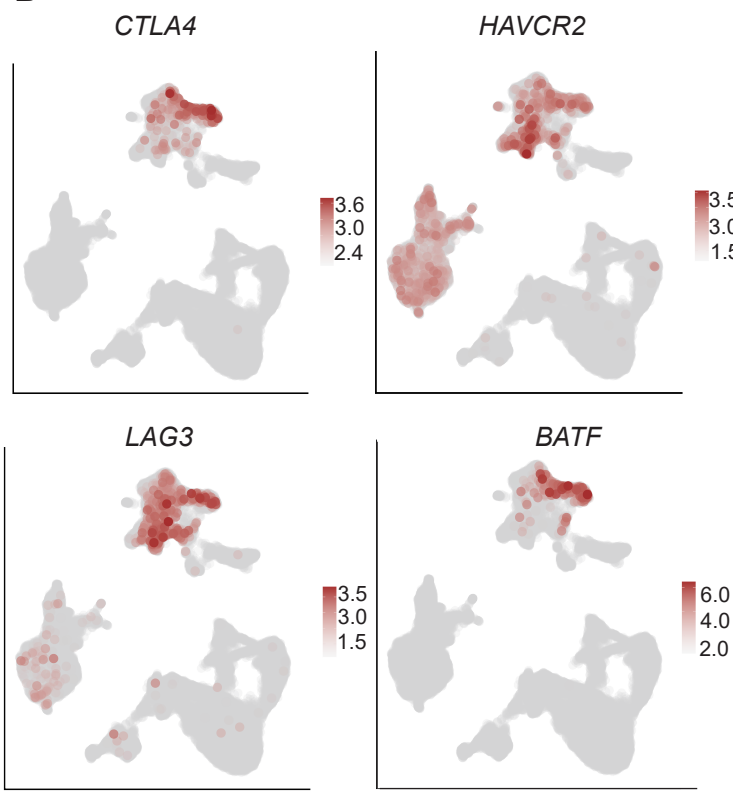

C

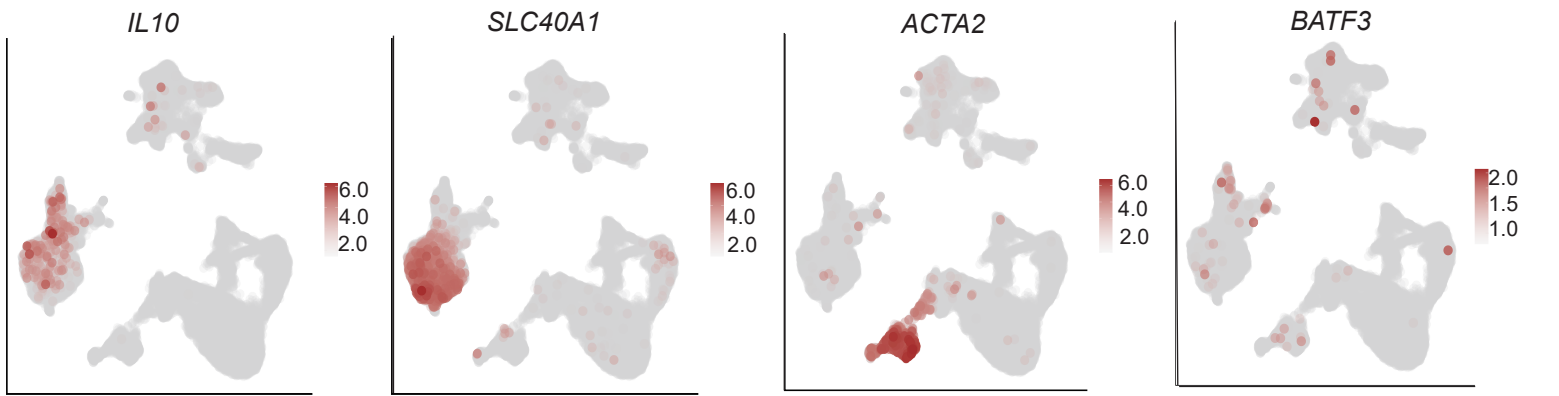

Supplement: Supplementary file 7 — Fig. S7. Expression of marker genes in GSE149614. (A) Violin plots showing the expression profile of marker genes in distinct subtypes (mMφ: 1283, myCAF: 1548, End: 1850, tT: 1128, aNK: 350, mT: 2763, tT: 1128, pT: 516, aT: 1600, nB: 1436, mB: 409, H1: 9855, H2: 1143, H3: 821, H4: 2733). In the violin plot, the centerline represents the median and box limits represent upper and lower quartiles; whiskers, data range. (B) Immune gene expression profile in the single‐cell RNAseq cohort GSE149614, colored from gray to red indicating the expression level from low to high. (C) Stromal gene expression profiles in the single‐cell RNAseq cohort GSE149614, colored from gray to red, indicating the expression level from low to high. [file MOL2-16-2195-s008.pdf]

**Figure S8**

**A**

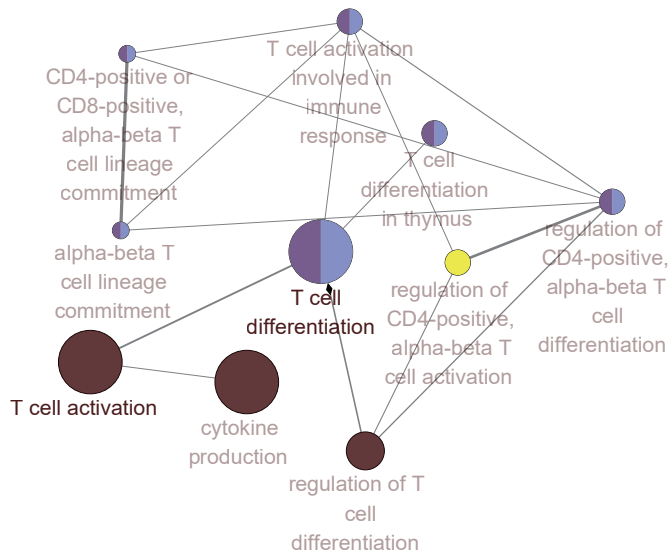

**B**

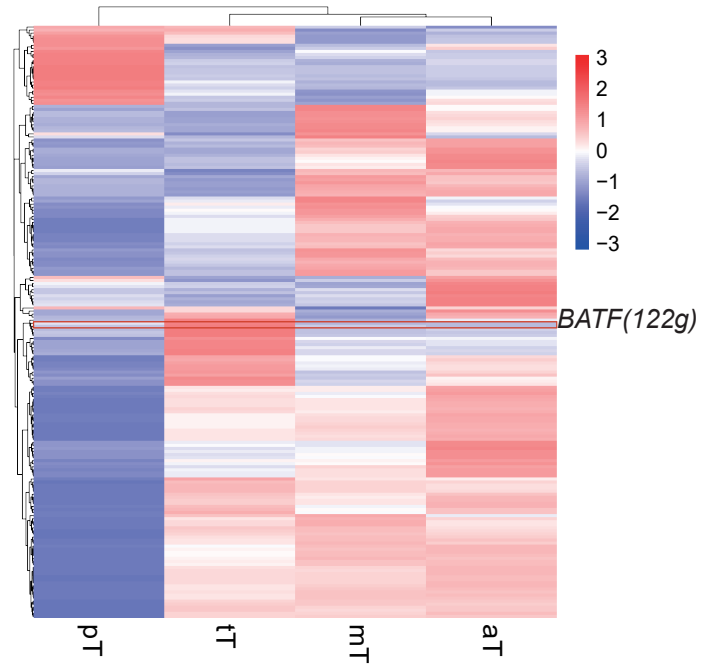

**C**

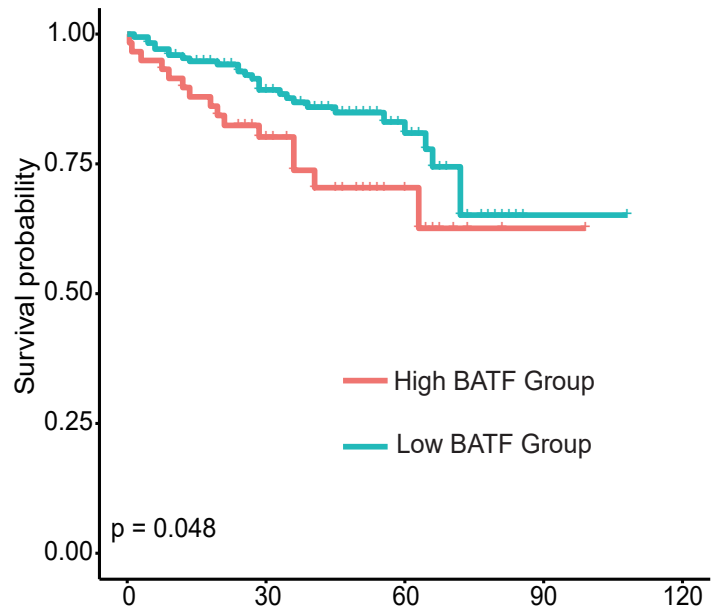

**D**

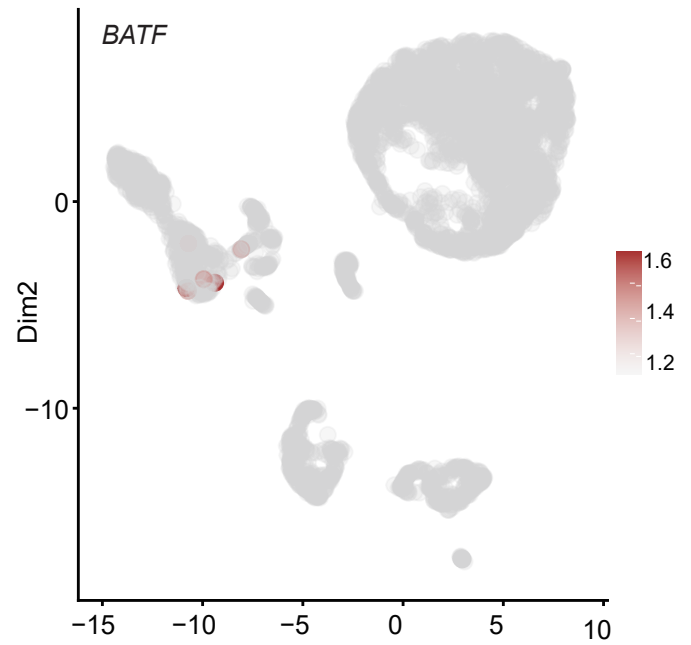

Supplement: Supplementary file 8 — Fig. S8. Gene enrichment and validation of the function of BATF in GSE149614. (A) Gene enrichment result in mT subtype (mT). (B) Heatmap showing the specificity of TF activation scores in the four T cell subtypes calculated by SCENIC. Color from blue to red indicates TF activation scores from low to high. (C) Overall survival curves showing the prognosis result of TF BATF in the ICGC cohort. Red and blue color indicates patients with a high expression level of BATF and low level in this cohort. The grouping cutoff value was calculated by X‐tile. Statistical significance was calculated using the log‐rank test (BATF‐High: 59, BATF‐Low: 173). (D) Expression of critical TF BATF in healthy human sample. Color key from white to red indicates the gene expression level from low to high. [file MOL2-16-2195-s007.pdf]

**Figure S9****A**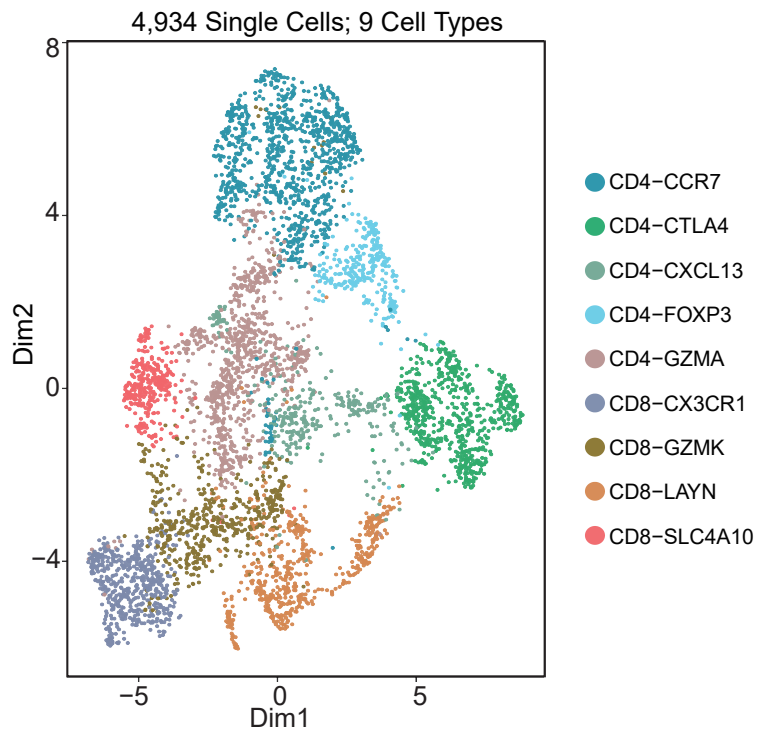**B**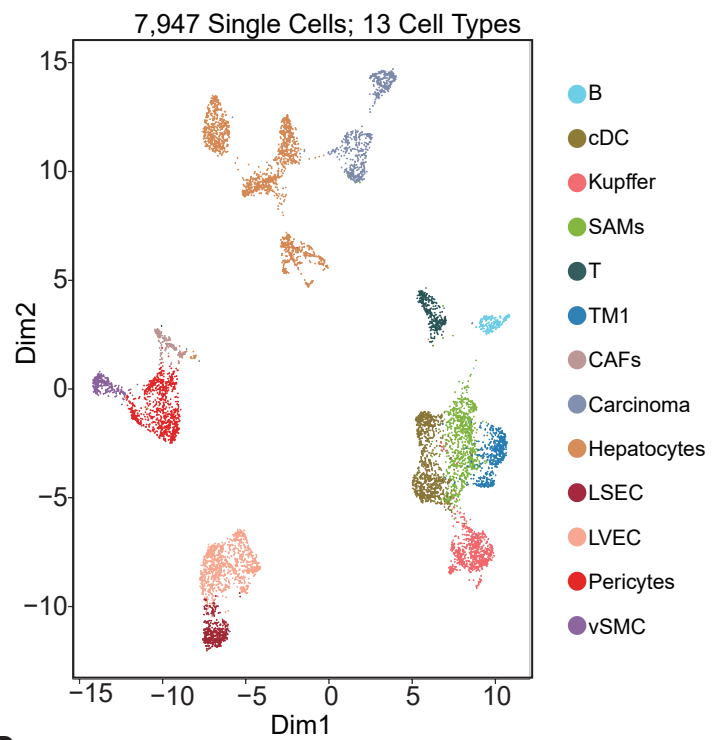**C**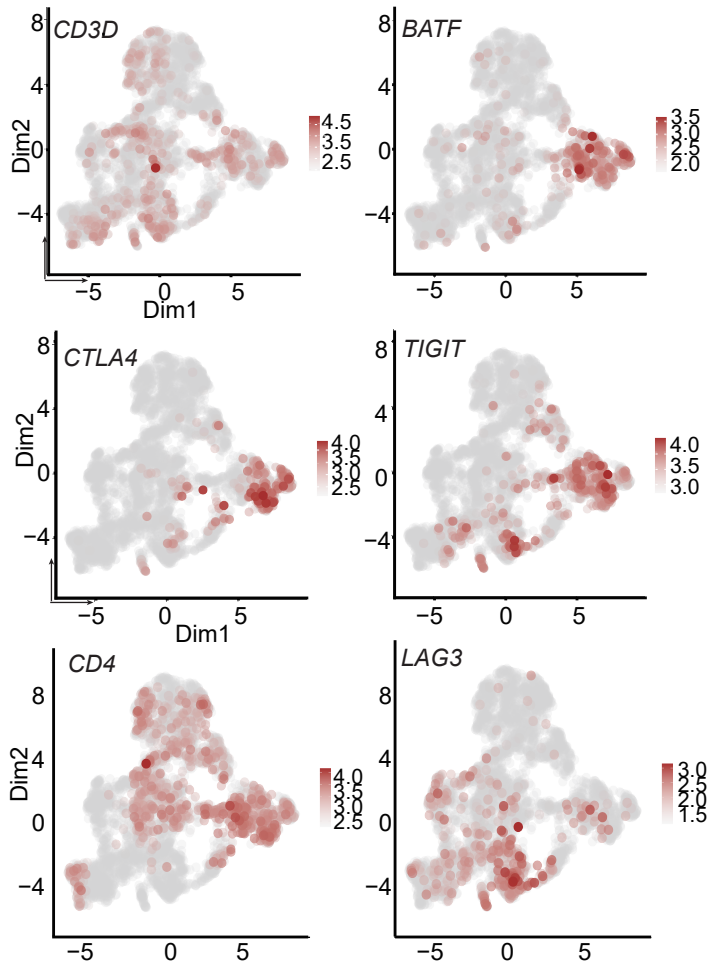**D**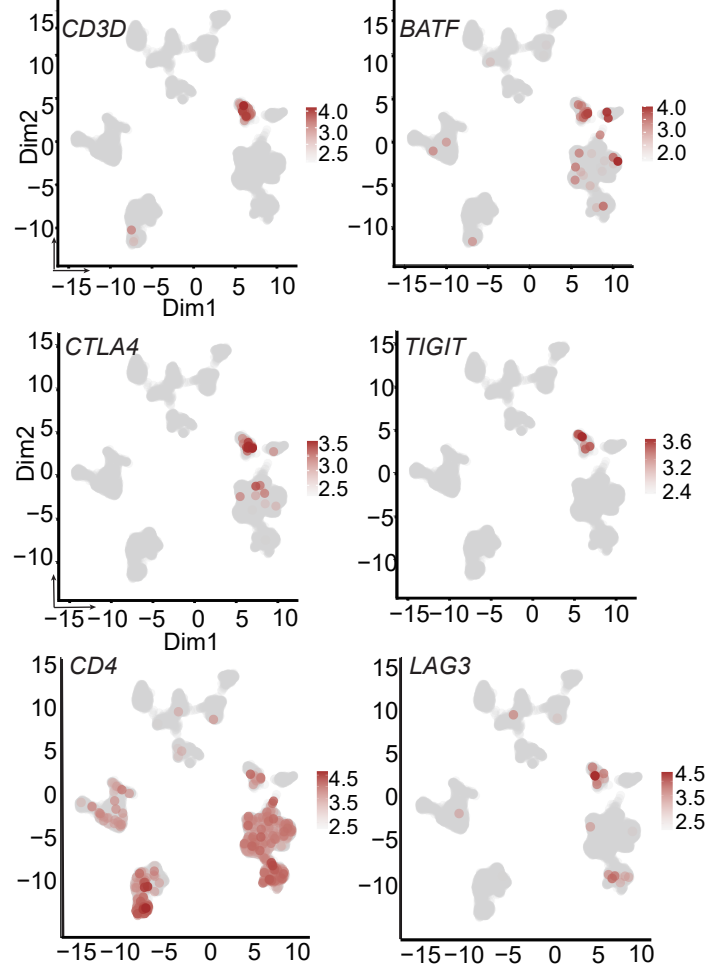**E**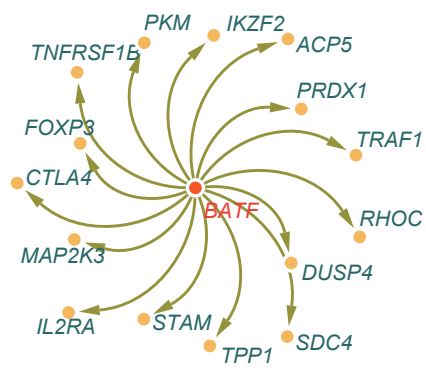**F**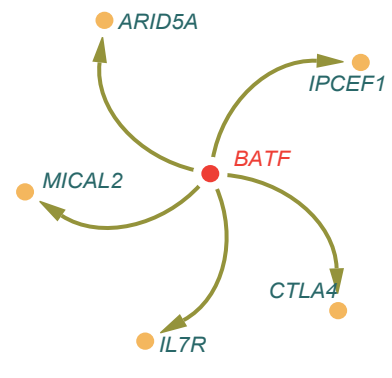

Supplement: Supplementary file 9 — Fig. S9. Expression of BATF and regulon in the other two single‐cell datasets. (A) The UMAP showing the profile of 4934 cells from single‐cell RNAseq dataset GSE98638. Clusters are indicated by different colors. (B) The UMAP showing the profile of 7947 cells from single‐cell RNAseq dataset GSE146409. Clusters are indicated by different colors. cDC: classical DC; SAM: scar‐associated macrophages; TM1: tissue monocytes; CAF: cancer‐associated fibroblasts; LVEC: liver vascular endothelial cells; LESC: liver sinusoidal endothelial cells; vSMC: vascular smooth muscle cells. (C) Gene expression pattern of immune genes in the GSE98638. Color from white to red indicates the gene expression level from low to high. (D) Gene expression pattern of immune genes in GSE146409 dataset. (E) BATF‐regulon of immunosuppressive T cells in GSE98638, in which red node indicates TF and yellow ones indicate target genes. (F) BATF‐regulon of immunosuppressive T cells in GSE146409, in which red node indicates TF and yellow ones indicate target genes. [file MOL2-16-2195-s004.pdf]
